# American Dreaming? Evaluating Trait Explanations for Health Inequalities by Race and Socioeconomic Status

**DOI:** 10.1177/00221465251368347

**Published:** 2025-10-23

**Authors:** Bruce G. Link, Ezra S. Susser, Pam Factor-Litvak, Barbara A Cohn

**Affiliations:** 1 University of California Riverside, Riverside, CA, USA; 2 Columbia University, New York, NY, USA; 3 New York State Psychiatric Institute, New York, NY, USA; 4 Child Health and Development Studies, Public Health Institute, Oakland, CA, USA

**Keywords:** health inequalities, life course, socioeconomic status, trait explanations

## Abstract

In the context of strong cultural beliefs associated with the American Dream, a prominent body of scholarship asserts that traits such as intelligence, self-confidence, and self-control play a pivotal role in the life course emergence of health inequalities by race and socioeconomic status (SES). We use prospectively ascertained data from approximately 50-year-old Black and White study participants (N = 605) whose mothers were recruited when pregnant with the participant. Follow-up into adulthood provided measures of cognitive and noncognitive traits, SES, and health needed to test trait explanations. Results show no evidence that traits are independently associated with adult health when SES is controlled or that they account for race or SES inequalities in health. Although the American Dream emphasizes individual traits as key factors shaping life outcomes, our results suggest the need to look elsewhere to understand why health inequalities by race and SES are such prominent social facts.

Is it possible that health inequalities by race and socioeconomic status (SES) are powerfully influenced by traits developed early in childhood? Traits such as intelligence, self-confidence, self-control, dependability, and a can-do spirit? An influential body of scholarship, aligned with deeply embedded American Dream ideals, makes two essential claims supporting trait-based explanations for health inequalities. The first has been debated for quite some time and asserts that traits are critical for educational and economic success—they lead to higher socioeconomic attainment. The second is that good health can be achieved and disease avoided by exercising such beneficial traits in health-relevant circumstances across the life course—they lead to better health (Calvin et al. 2011; Conti and Heckman 2010; Deary 2020; Gottfredson 2004; Heckman 2006, 2013; Oi and Alwin 2017; Wraw et al. 2015). If both claims are true and the effects of traits are strong enough, a substantial portion of observed health inequalities by race and SES might be accounted for by differences in the development and deployment of such traits. For socioeconomic inequalities, the argument is that because cognitive and noncognitive traits are developed early in life, they lie anterior to both adult SES and adult health, causing them both. In this scenario, a large portion of the association between adult SES and adult health is rendered spurious when traits are controlled. For racial inequalities, the claim is that early life deficits in cognitive and noncognitive traits are more common among racial minorities and are a key driver of subsequent socioeconomic and health inequalities.

Interestingly, U.S. nationwide data from the Rand Corporation indicate that people believe that both health (71.2%) and educational attainment (63.4%) are the result of the “choices” a person has made. And when asked to list three reasons “why people with lower incomes live 7.5 years less than people with higher incomes,” 50.3% list “personal choices.” By contrast, “treatment by society of those with low incomes” and “discrimination” were mentioned only by 15.1% and 8.3%, respectively (Carman et al. 2019). “Choices” implicate individual decision-making, and lying behind such choices are traits such as self-control, self-efficacy, and intelligence. In sum, because strong claims about the importance of traits for health inequalities have been made in the research literature and because large segments of the American public identify the sources of such inequalities in ways that are consistent with trait explanations, we propose an analysis that tests such trait-based claims. Our goal, therefore, is to test whether cognitive and noncognitive traits have effects on health that are independent of SES and play a substantial role in explaining health inequalities by race and SES.

As we turn our focus toward trait explanations, it is important to keep in mind the large body of research that has signaled the importance of the adversity associated with low SES and systemic racism in the emergence of health inequalities across the life course (Brady et al. 2022; Brown and Homan 2024; Ferraro, Schafer, and Wilkinson 2016; Gee and Hicken 2021; Hayward and Gorman 2004; Hicken, Lee, and Hing 2018; Phelan and Link 2015). Studies have, for example, focused on early life adversity to examine whether such experiences carry through to influence exposure to adult adversities and adult health (Ferraro et al. 2016; Hayward and Gorman 2004; Kim et al. 2021; Montez and Hayward 2011; Umberson 2017; Umberson, Crosnoe, and Reczek 2010). Studies have also underscored the importance of adult life circumstances, often finding that these adult life circumstances plausibly mediate early life conditions in their effect on adult health (Cha, Farina, and Hayward 2021; Donnelly 2022; Ferraro et al. 2016; Hayward and Gorman 2004). In this accounting, socioeconomic disadvantage and racism induce stress (e.g., Geronimus et al. 2006) and deplete flexible resources (e.g., Link and Phelan 1995; Phelan and Link 2015) to harm health. Notwithstanding this body of research, trait explanations have been proposed that essentially provide a counternarrative to it. The current study does not provide new evidence regarding the role of adversity in health inequalities but instead takes up the challenge of this counternarrative by implementing a test of its truth value.

To investigate trait explanations for health inequalities, life course data from early in life into adulthood are required. They are, however, very rare, especially in the multiracial context of the United States. We report evidence from a follow-up study that we conducted of approximately 50-year-old Black and White adults (N = 605) whose mothers were recruited into the Child Health and Development Studies (CHDS) when they were pregnant with the participant. Importantly, the data include measures of cognitive and noncognitive traits, SES, and health across the life course to allow a prospectively achieved test of trait explanations for health inequalities.

## Background

### Trait Explanations for Health Inequalities: Theory and Evidence

The idea that traits such as intelligence, self-control, self-confidence, and dependability can be effectively exercised to achieve social and economic success is deeply embedded in American culture. It is an important element of the “American Dream” idea that indicates that it is through the exercise of such traits that anyone in the United States can rise to enjoy a successful life. Credited as its originator, Adams (1931:xvi) indicated that the American Dream consisted of the idea that “life should be better and richer and fuller for everyone, with opportunity for each according to ability or achievement.” The *Oxford English Dictionary* (2025) defines the American dream as “the ideal that every citizen of the United States should have an equal opportunity to achieve success and prosperity through hard work, determination, and initiative.” Traits—ability, determination, initiative—are what make the desired success achievable. The connection to social inequalities in health emerges from the argument that these traits are not only important for economic success but also play a substantial role in shaping health outcomes—directly influencing health and thereby explaining a substantial portion of the health inequalities observed across racial and socioeconomic groups.

#### Intelligence

The idea that intelligence is critical in health inequalities was strongly proposed by Gottfredson (2004:174), who suggested that intelligence could be the “epidemiologists’ elusive ‘fundamental cause’ of social class inequalities in health.” The rationale for this claim is that intelligence can be operationally defined as a common core trait that is useful for grasping concepts, retaining information, and performing with accuracy and efficiency. Certainly, people who score well on tests measuring it reliably attain higher levels of education (Deary 2020). But, the argument continues, intelligence not only leads to educational attainment but also independently influences health. The idea is that crafting a healthy lifestyle is a complex task and that people who are more adept in this domain are better able to access information, weigh evidence, and then construct a successful plan to address the health circumstances that they confront. According to this argument, adequate control of intelligence could “explain” much of the race–health association and/or render a substantial portion of the SES–health association spurious.

Evidence pertaining to claims about the importance of intelligence comes from the so-called “cognitive epidemiology” literature that includes studies of individuals assessed for intelligence relatively early in life and then followed into adult life for health outcomes. A meta-analysis of 16 studies led to the conclusion that a 1 *SD* unit advantage in intelligence quotient (IQ) was associated with a 24% decrease in mortality risk and that controls for childhood SES showed no evidence of confounding the IQ–mortality association (Calvin et al. 2011). Although both the Calvin et al. (2011) review and research conducted since then (Deary, Hill, and Gale 2021) report an IQ–health association, the control of life course SES was consistently weak, usually involving only a single indicator assessed at a single time and focused only on paternal indicators of SES circumstances (Link et al. 2017).

Another pattern of evidence in the cognitive epidemiology literature is that controls on indicators of adult SES often reduce the association between measures of intelligence and health to nonsignificance. For example, in a study using the U.S. National Longitudinal Study of Youth, Wraw et al. (2015) found significant associations between the Armed Forces Qualifications Test at ages 14 to 21 and multiple self-reports of illness conditions at age ≈50. However, when adult SES is controlled, associations are substantially reduced, and most (8/10) are rendered nonsignificant. Many studies show this same pattern (Calvin et al. 2011; Clouston et al. 2015; Link et al. 2008, 2017) and therein challenge the claim that cognitive ability has an independent effect on health. Despite this, claims about the role of cognitive abilities endure either by dismissing the role of education as only a proxy for intelligence (Wraw et al. 2015) or by implementing only very weak measures of adult SES.

#### Noncognitive traits

The claim that traits such as self-control, dependability, and self-confidence are important in the creation of health inequalities has been prominently advanced by multiple investigators (Carter et al. 2019; Heckman 2006, 2008, 2013; Heckman and Mosso 2014; Oi and Alwin 2017). For Heckman and colleagues (Heckman, Stixrud, and Urzua 2006:413–14), the core argument downplays the role of socioeconomic circumstances in asserting that noncognitive traits are instilled by “more able and engaged parents” and that “because cognitive and noncognitive abilities are shaped early in the life cycle, differences in these abilities are persistent, and both are crucial to social and economic success; gaps among income and racial groups begin early and persist.” Following this reasoning, Heckman (2013:40) concludes that the “proper measure of child adversity is the quality of parenting—not the traditional measures of family income or parental education.” Deficits in the resulting traits are seen as broadly disserviceable as they determine “labor market outcomes, the likelihood of marrying and divorcing, the likelihood of receiving welfare, voting, and health” (Heckman and Mosso 2014:691). Sociologists Oi and Alwin (2017:198) concur, emphasizing that traits are central to socioeconomic inequalities and have “profound influences on both education and health in early adulthood and that a substantial portion of the latter association is spurious.”

The mechanistic explanation linking noncognitive traits to economic success and health is that the more planful, motivated, confident, self-controlled, and conscientious a person is, the better able they are to develop the knowledge and skills needed for educational and occupational success. These traits are also essential for creating a healthy lifestyle. For example, a person must plan how to exercise, what to eat, and when to sleep and then conscientiously stick to the plan and have the self-control not to stray from it.

Pertaining to evidence concerning noncognitive traits, two small early life experimental interventions with disadvantaged children—the Abecedarian and Perry studies—have been cornerstones of the noncognitive approach because they demonstrated long-term impacts across many outcomes, including health, that were presumed to have been mediated by cognitive and noncognitive traits (Campbell et al. 2014; Heckman, Pinto, and Savelyev 2013). However, evidence that the experimental intervention changed noncognitive traits and that these changes are what led to the beneficial long-term outcomes is either absent (Abecedarian) or weak and inconsistent (Perry; see Supplement Section 1 in the online version of the article).

Other evidence comes from a meta-analytic review of noncognitive traits (Smithers et al. 2018). The review concludes that evidence for the long-term effects of such traits on multiple outcomes, including health, is lacking. With respect specifically to health, the authors found conclusions “difficult to draw” and reported only modest effect sizes for noncognitive traits on health (range = .06–.14). Additionally, most studies investigating health involved relatively short-term follow-ups (median follow-up of 4.2 years), suggesting the need for longer-term follow-up studies like ours.

Finally, two studies not included in the Smithers et al. (2018) review provide long-term follow-up of prospectively collected noncognitive traits in relation to SES, one using the 1970 British Cohort study (Oi and Alwin 2017) and the other the 1958 British Cohort study (Carter et al. 2019). Although the analyses produce useful data points, they are based on homogeneously White populations, are restricted to self-report health outcomes, and in the case of Oi and Alwin (2017), report follow-up only to age 30. However, the most consequential issues involve the life course measurement of SES because both studies rely on dichotomized measures of what are known to be finely graded associations and neither study included indicators of income or wealth (see Supplement Section 2 in the online version of the article).

### Aims of the Current Research

The core issue that we address is whether cognitive and noncognitive traits have direct effects on health that are both independent of comprehensively assessed SES and play an important role in accounting for health inequalities by race and SES. Although traits may play other roles in shaping people’s life chances beyond health, these are not an issue in our inquiry. For example, we do not challenge the potential role cognitive and noncognitive traits may play in influencing the attainment of education or income. Instead, we are interested in examining the claimed potency of such traits in accounting for the emergence of health inequalities across the life course. According to trait explanations for health inequalities, traits lie anterior to the life course attainment of SES and adult health, serving as a common cause of both. Prospectively collected data from across the life course and into adult life that might test such claims are relatively rare. The current study provides (1) multiply operationalized child assessments of cognitive abilities (ages 9 and 15–17), (2) multiple relevant noncognitive traits assessed by mother and self-report, and (3) prospectively ascertained SES from birth through adolescence. Importantly, the data also include self-report and biomarker assessments of health and multiply operationalized measures of SES assessed at age ≈50 in Black and White study participants. Taken together, these characteristics of the current study provide a strong setting in which to test trait explanations for health inequalities.

Table 1 presents four patterns of results that could emerge. The first row shows a pattern that would support trait explanations for health inequalities because traits are unequally distributed by race and independently associated (net of relevant controls) with adult SES (Column 1) and adult health (Column 2). Then, because of these associations, adult SES and race are no longer independently associated with health when traits are controlled (Column 3). Rows 2 through 4 show ways that trait explanations might fail to explain health inequalities. Traits could fail because there are no differences in traits by race and because such traits are not independently related to adult SES (Row 2). They could also fail if they are not independently associated with adult health (Row 3) or with both adult SES and adult health (Row 4). Although we provide evidence regarding all predictions in Table 1, it is the independent effect of traits on health (represented in Rows 3 and 4) that will prove most critical. If traits are to explain substantial proportions of SES and race inequalities in health, such independent associations must be observed.

**Table 1.** Patterns of Results That Would Support or Fail to Support Cognitive and Noncognitive Trait Explanations for Adult Health Inequalities.

	Column 1	Column 2	Column 3
	Traits Are Independently Associated with Race and Adult SES	Traits Are Independently Associated with Adult Health^a^	Race and Adult SES Are Independently Associated with Adult Health^b^
Row 1: Supports trait explanations	Yes	Yes	No
Row 2: Fails to support trait explanations because traits are not independently associated with race and adult SES	No	Yes	Yes
Row 3: Fails to support trait explanations because traits do not independently predict health	Yes	No	Yes
Row 4: Fails to support trait explanations because traits do not independently predict either race/adult SES or health	No	No	Yes

*Note:* SES = socioeconomic status.

a Controlling race, child SES, and adult SES.

b Controlling for all traits and child SES.

## Methods

### The Child Health and Development Studies Cohort

Participants were the adult offspring of families that were originally recruited as part of the Child Health and Development Studies (2025). Virtually all pregnant women (19,044 live births) receiving prenatal care from the Kaiser Foundation Health Plan at its facilities in Alameda County, California, were recruited between 1959 and 1967. Subsets were selected and recruited for participation in follow-up assessments, including interviews with mothers at offspring ages 5, 9 to 11, and 15 to 17 and with offspring at ages 15 to 17.

### The CHDS Disparities Study Sample

The CHDS disparities study collected data on cohort members when they were approximately 50 years old using a 40-minute computer-assisted telephone interview, a home visit, and a self-administered questionnaire between May 2010 and October 2012. We aimed to enroll 350 non-Black and 250 Black participants, evenly distributed by sex assigned at birth. Participants were recruited from a pool of 3,196 eligible individuals who had taken part in the CHDS child follow-up studies. We limited participants to current residents of California (79% of the eligible pool) due to the difficulty and cost of implementing home visits nationwide. We selected 1,633 individuals to locate and include in our study. They comprised (1) a 50% random sample of non-Black male and female participants in the CHDS Adolescent Study, (2) 100% of Black male and female participants in the CHDS Adolescent Study, and (3) to achieve an adequate sample of Black participants, a supplementary group of 100% of Black male and female participants in the CHDS follow-up studies at age 5 and/or ages 9 to 11 who were not assessed at age 15 to 17. We obtained phone numbers for 1,073 of the selected pool and achieved target sample sizes after contacting 985—a 61% survey participation rate. Of the 605 individuals who participated in the telephone interview, 510 (84%) completed a home visit, and 400 provided blood for biomarker data (additional details in Supplement Section 3, Table S1, and Figure S1 in the online version of the article).

Potential participants who moved out of California did not differ significantly from those who stayed on mother’s age, education, and employment; father’s age, education, and employment; family income; and offspring birthweight in either Black or White participants. We also compared cohort members who eventually participated to cohort members who did not on these same variables and once again, found no significant differences (see Table S2 in the online version of the article).

To provide further evidence, we compared CHDS disparities data to the nationally representative National Health and Nutrition Examination Survey (NHANES; National Center for Health Statistics 2013). Using NHANES participants assessed during the same period (2011–2012) and in the same age range (46–52), we found no significant overall differences between our sample and NHANES for body mass index, the biomarker hemoglobin A1C, systolic and diastolic blood pressure, or self-rated health. Additionally, we found that disparities by race and SES (education) on these variables in our data were generally also present in NHANES. Importantly, in no instance was there a race or educational health inequality that was significantly stronger in one sample than in the other. The fact that our sample contained the same health inequalities as NHANES suggested its utility as a place to test the role of cognitive and noncognitive traits as a source of such inequalities (see Supplement Section 4 and Tables S3 and S4 in the online version of the article).

### Measures

Measures were from archived data collected during childhood and from the current study at age ≈50. All dummy-coded variables were scored 0 = absent or 1 = present. Continuous predictor variables were scored such that the lowest score was 0 and the highest 1 and all other values arrayed continuously between. This coding rendered the magnitude of each coefficient as the difference between the highest scoring participant and the lowest scoring participant. Outcome variables remained in their original metric.

*Adult self-rated health* (SRH; n = 603) was assessed in midlife in both the telephone survey and the self-administered leave-ahead by asking, “In general, would you say your health is excellent (5), very good (4), good (3), fair (2), or poor (1)?” The score analyzed was the average of the two measures (α = .87).

*Allostatic load* (AL; n = 400) is a concept that refers to the physiological dysregulation of bodily systems due to chronic stress (McEwen and Stellar 1993). Our measure included 10 biomarkers: percentage body fat, waist circumference, high-density lipoprotein (HDL) cholesterol, the ratio of total to HDL cholesterol, hemoglobin A1C, C-reactive protein, interleukin-6, systolic and diastolic blood pressure, and dehydroepiandrosterone sulfate (see Kezios et al. 2022; also see Supplement Section 5 in the online version of the article). We constructed an index of AL in which each biomarker is dichotomized, with scores in the highest risk quartile scored as 1 versus 0 otherwise.

*Race-ethnicity* (n = 605) was based on participant self-identification in adult life and grouped into non-Hispanic White (n = 310, 51.2%), non-Hispanic Black (n = 244, 40.3%), and because of small numbers, other race-ethnicity (n = 51, 8.4%; for more detail, see Supplement Section 5 in the online version of the article).

*Sex at birth* (n = 603) was ascertained from the archived data (female = 1, male = 0), with respondents asked at age 50 whether they considered the assignment accurate; all participants indicated that it was.

*Childhood SES* (n = 603) included three equally weighted prospectively collected indicators: (1) maternal education at birth; (2) paternal occupation at birth, 9 to 11, and 15 to 17 follow-ups; and (3) mother-reported family income also assessed at birth, 9 to 11, and 15 to 17. Because maternal education was assessed in categories, we scored it 0 = less than high school (n = 53, 9%), .33 = high school graduate (n = 187, 31%), .67 = some college (n = 123, 20%), and 1 = college or more (n = 114, 19%). As described in Section 5 of the Supplement in the online version of the article, we constructed scores for paternal occupation and family income by averaging each across the three times they were assessed. We then created a composite measure based on the mother’s education, father’s occupation, and family income that ranged from 0 to 1.

*Adult SES* (n = 603) was a composite measure, consisting of indicators of education, occupational standing, family income, and wealth. We decided on a composite measure because we are interested not only in what is unique to each indicator but also in what is shared by them. Education was assessed by asking about years of regular schooling, occupation by classifying current or last occupation to three-digit census categories and applying scores from Nam-Powers-Boyd Occupational Status Scores (2022), income by asking about total family income using 12 categories ranging from $2,500 or less to $300,000 or more, and wealth by ascertaining assets such as home value, bank accounts, and stocks and bonds while subtracting liabilities (e.g., mortgages). The top score for each indicator was assigned a score of 1 and the bottom score a 0, with all other scores arrayed in between. The composite score was constructed by averaging the four 0 to 1 indicators to yield an overall continuous score of 0 to 1. Our approach adopted a “relative” versus “absolute” assessment by assigning a relative rank between 0 and 1 to each individual rather than absolute income, education, occupation, and wealth (Brady et al. 2023) . In a supplemental analysis (Supplement Section 5, Tables S11 and S12 in the online version of the article), we separately enter each dimension of SES measured in absolute terms, finding no change in our conclusions regarding trait explanations for health inequalities.

*Cognition* (n = 554) was assessed using two commonly used measures, the Peabody picture vocabulary tests (Dunn and Dunn 1959), administered at 9 to 11 and 15 to 17, and the Raven progressive matrices, administered at 9 to 11(Raven 1989). We used all available tests to construct a composite measure of cognitive ability (462 had all three tests, 88 had two tests, 4 had one test, and 51 had no tests). We constructed a composite because entering each one separately would be, we reasoned, too stringent a test because each would be partialed for the other, demanding that each one uniquely accounts for the outcomes to attain significance (for a sensitivity analysis that does not use a composite, see Supplement Section 5 in the online version of the article). We recoded to assign the lowest score 0 and the highest 1, with all other scores arrayed continuously in between. Cognitive scores strongly predicted subsequent college graduation (odds ratio of 7.4 associated with a 2 *SD* difference). The multioperationalization of cognition with different but well-established tests, measured at different times, plus the robust prediction of educational attainment suggested a strong measurement platform for assessing the importance of cognition in explaining health inequalities.

### Noncognitive Traits

*Trustworthy/self-controlled* (n = 456) was assessed at 9 to 11 by asking mothers to rate their offspring on multiple characteristics. The 20 ratings that we selected were representative of what are called “externalizing symptoms” in the fields of child development and psychiatric epidemiology (Achenbach et al. 2016) and were used by Heckman et al. (2013) as noncognitive traits. Mothers were asked if a characteristic was “true” (scored 3) or “not true” (scored 1) of their child or whether the mother was “uncertain” (scored 2). Youth could be rated as trustworthy/untrustworthy (e.g., ratings of truthful, dependable, steals), self-controlled/not self-controlled (e.g., daredevil, temper explosions, starts fights), and cooperative/uncooperative (e.g., minds without a fuss, sassy talks back, stubborn), capturing core components of what the noncognitive traits literature refers to when it defines the construct. Items like these are commonly operationalized as a single externalizing factor, in contrast to an internalizing factor—an approach also supported by an exploratory factor analysis of our data. The analysis indicated a single-factor solution (first eigenvalue = 5.09; second = .72), leading us to construct a single scale (α = .86). For more information about this measure, see Supplement Section 5 in the online version of the article.

Pearlin and Schooler’s (1978) *mastery* (n = 468) and Rosenberg’s (1989) *self-esteem* (n = 469) were ascertained at age 15 to 17. They fit the noncognitive framework in that mastery captures a “can-do attitude” and self-esteem captures “confidence.” Both the 7-item measure of mastery and the 10-item measure of self-esteem asked the degree of agreement using a 4-point strongly agree (1) to strongly disagree (4) format and then combined the items to create scale scores (mastery: α = .68; self-esteem: α = .79).

The measure of *valued noncognitive traits* (n = 468; α = .74) was assessed at 15 to 17 and asked how important it was for young people to have characteristics including facing life’s problems calmly, having self-control, being dependable, defending one’s point of view, and being considerate. Participants indicated how important each trait was on a 4-point scale ranging from extremely important (4) to not important at all (1).

Mastery and self-esteem were correlated .56, whereas other measures were only modestly correlated. Because measures (1) directly assessed core traits identified in the noncognitive literature, (2) involved a multioperationalization of such traits, (3) included assessments from both mothers and offspring, and (4) captured traits prospectively to allow long-term follow-up, they provided a strong platform for testing the importance of such traits in generating health inequalities.

*Child and adolescent health* (n = 568) was assessed using nine indicators of conditions, including gestational age, doctor-identified anomalies, high blood pressure, asthma, and others (see Supplement Section 5 in the online version of the article), that were collected at birth, age 5, and age 15 to 17. About half (50.6%) of children experienced none of the nine indicators, 35% experienced one, 10.1% experienced two, and only 4.3% experienced three or more.

*Family context* included marital separation or divorce (n = 603; 1 = yes, 0 = no), a six-item measure of respondent’s report of the quality of their relationship with their mother (α = .76; n = 473), and a retrospective question at midlife concerning whether a parent’s drug or alcohol use caused serious problems (n = 470) for the family (yes = 1, no = 0).

### Approach to Missing Data

The main reason that missing data occurred was that participants were not assessed at every wave during childhood. We addressed missing data using multiple imputation (Stata Corp, College Station, TX) for all but dependent variables. We chose multiple imputation because it uses all available information, whereas complete case analysis does not, and it allows us to use the same approach to missing data for both our multiple linear regression and negative binomial models. This resulted in 603 cases for the SRH outcome and 400 cases for the AL measure. Twenty-five data sets were imputed and recombined using Rubin’s (1987) rules. As a sensitivity analysis, we conducted a complete case analysis.

### Analysis

We used multiple regression to analyze self-rated health as a continuous variable and negative binomial regression to analyze AL as a count variable. We began by analyzing race and socioeconomic inequalities in adult SRH and AL. Next, we examined associations between cognitive and noncognitive traits and health outcomes. In keeping with Table 1, Column 1, we first tested whether traits were associated with adult SES and race. We then tested whether traits (1) were independently associated with health when controlling for race, childhood SES, adult SES, and other covariates (Table 1, Column 2) and (2) whether trait measures accounted for associations between race and health and adult SES and health (Table 1, Column 3). We reported how much race and adult SES associations decline as trait variables were entered, using the Stata-supported program PARAMED (Emsley and Liu 2013) in the analysis of AL.

## Results

### Adult Health Inequalities by Race and Socioeconomic Status

**Figure 1.**
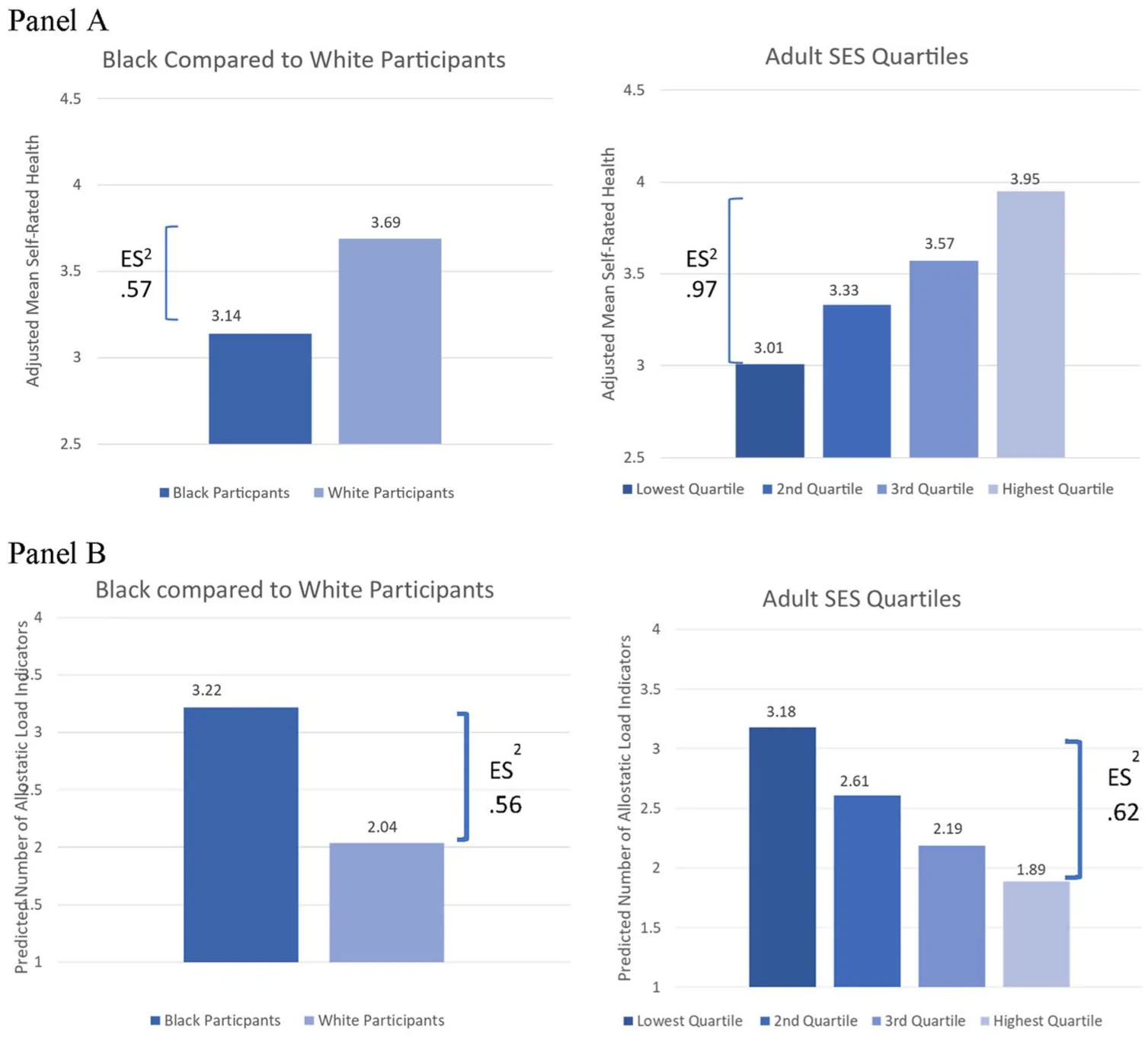
Age- and Sex-Adjusted Inequalities in Self-Rated Health and Allostatic Load by Race and Adult Socioeconomic Status: Child Health and Development Disparities Study. Panel A: Self-Rated Health (n = 603). Panel B: Allostatic Load (n = 400). *Note:* As indicated in the text, every association depicted is statistically significant. ES = effect size comparing gap between Blacks and Whites and the top and bottom quartile for SES, with each divided by the standard deviation of the health outcome. SES = socioeconomic status.

Multiple (SRH) and negative binomial (AL) regression analyses show highly significant age- and sex-adjusted health inequalities by race (SRH: *b* = –.546, *p* < .001; AL: *b* = .456, *p* < .001) and adult SES (SRH: *b* = 1.829, *p* < .001; AL: *b* = –1.068, *p* < .001). Figure 1 shows these effects graphically by quartiles of SES. Concerning SRH (Figure 1, Panel A), Black participants have a substantially lower adjusted mean SRH score (3.14) than White participants (3.69), a difference corresponding to an effect size of .57. Concerning adult SES, there is a stepwise gradient across SES quartiles, with the lowest quartile having significantly lower SRH (3.01) than the highest quartile (3.97), a difference corresponding to an effect size of .97. AL indicators (Figure 1, Panel B) are higher among Black participants (3.22) than White participants (2.04), with an effect size of .56, and concerning SES, a monotonic gradient shows a large gap between the lowest (3.18) and highest quartiles (1.89), with an effect size of .62. Tables S5 to S7 in the online version of the article show that the overall pattern of race and adult SES health inequalities described here is also evident within sex and racial groups.

### Do Cognitive and Noncognitive Traits Predict Self-Rated Health and Allostatic Load?

**Figure 2.**
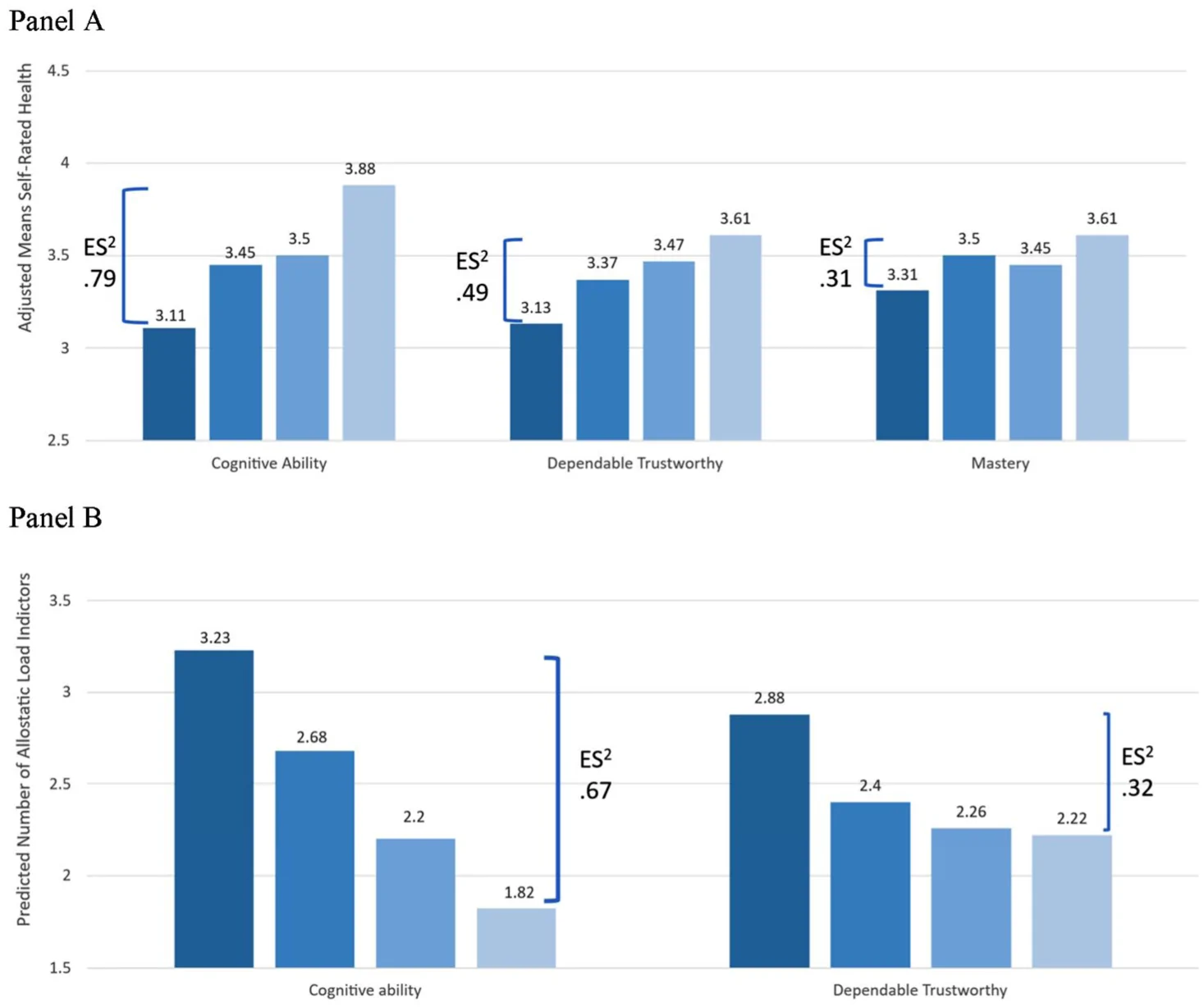
Age- and Sex-Adjusted Inequalities in Self-Rated Health and Allostatic Load by Cognitive and Noncognitive Traits: Child Health and Development Disparities Study. Panel A: Age- and Sex-Adjusted Means of Self-Rated Health by Quartiles of Trait Measures (n = 603). Panel B: Age- and Sex-Adjusted Predicted Number of Allostatic Indicators by Quartiles of Trait Measures (n = 400). *Note:* As indicated in the text and shown in Table S8 in the online version of the article, each trait is significantly associated with the health outcome in question. ES = effect size comparing gap between the top and bottom quartile divided by the standard deviation of the health outcome.

Adult SRH was significantly predicted by childhood trait measures of cognitive ability, trustworthy/self-controlled, and mastery, whereas AL was significantly predicted only by cognitive ability and trustworthy/self-controlled. Table S8 in the online version of the article shows these results with all traits entered as continuous variables, and Figure 2 depicts them graphically by showing the health outcomes by quartiles of the traits that were significantly associated with the health outcomes. As Figure 2 shows, gaps in SRH between the lowest and the highest quartile were 3.11 versus 3.88 for cognitive ability (effect size = .79), 3.13 and 3.61 for trustworthy/self-controlled (effect size = .49), and 3.31 and 3.61 for mastery (effect size = .31). Gaps between the lowest and the highest quartiles concerning the predicted number of AL indicators are 3.32 and 1.82 for cognitive ability (effect size = .67) and 2.88 and 2.22 for trustworthy/self-controlled (effect size = .32). Thus, 5 of the possible 10 childhood-assessed cognitive and noncognitive trait indicators significantly predicted adult health. These results suggest a potential role for traits in adult health but do not represent strong support because we do not yet know whether the effects are independent of SES, race, and other factors because they would have to account for health inequalities by race and adult SES.

### Are Traits Associated with Race and Socioeconomic Attainment?

As depicted in Table 1 (Column 1), traits need to be independently associated with race and adult SES to plausibly account for health inequalities. For adult SES, the issue is whether increased levels of cognitive and noncognitive traits are associated with higher levels of adult SES. For race, the issue is whether racial minorities experience lower levels of cognitive and noncognitive traits and because of this, experience untoward outcomes, including health problems in adult life.

**Figure 3.**
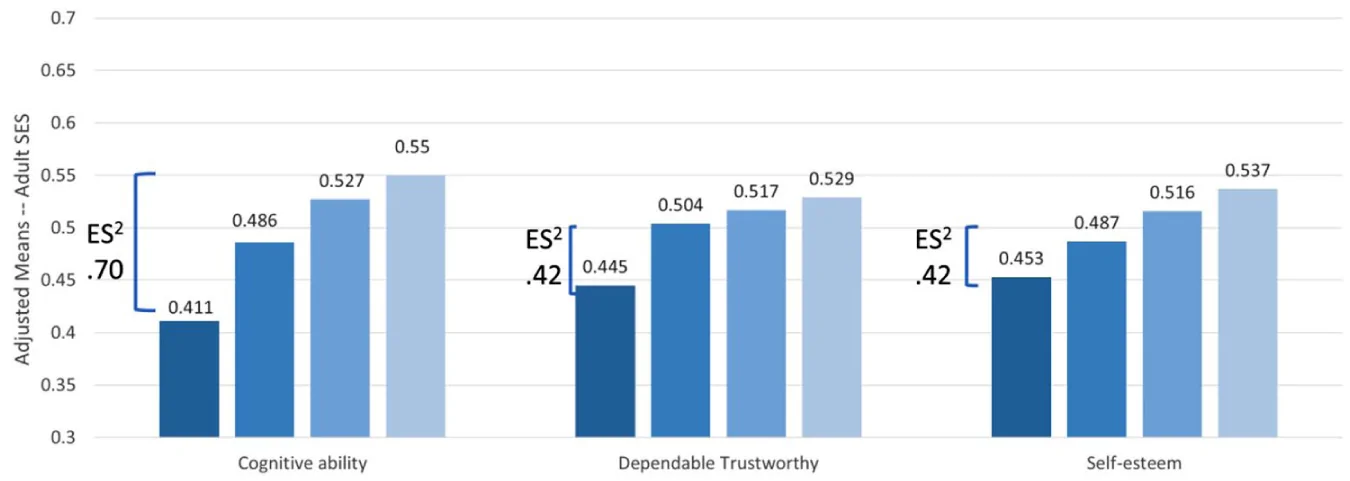
Adjusted Means of Adult SES by Quartiles of Cognitive and Noncognitive Traits. *Note:* As indicated in the text and shown in Table S8 in the online version of the article, each trait shown is significantly associated with adult SES controlling for age, sex, childhood SES, and self-identified race ethnicity. ES = effect size comparing gap between the top and bottom quartile divided by the standard deviation of adult SES. SES = socioeconomic status.

Consistent with trait explanations, Table S9 in the online version of the article shows that all five of the traits predict adult SES while controlling for age, sex, race-ethnicity, and childhood SES. When all five traits are entered together, three—cognitive ability, trustworthy/self-controlled, and self-esteem—are independently significant. Figure 3 depicts the trait to adult SES relationships graphically by showing adult SES within quartiles of the traits. Effect sizes comparing the average adult SES in the lowest quartile of each trait to the average adult SES in the highest quartile range from a high of .70 for cognitive ability to a low of .23 for mastery, with trustworthy/self-controlled (effect size = .42), self-esteem (effect size = .42), and valued noncognitive traits (effect size = .24) lying in between.

Concerning associations between race and the traits, Table S10, in the online version of the article, provides a more mixed picture. Consistent with a trait explanation for health inequalities, cognitive scores and mother-rated trustworthy/self-controlled are significantly lower in Black participants than in White participants with age, sex, and childhood SES controlled. However, measures of valued noncognitive traits (nonsignificant), mastery (nonsignificant), and self-esteem (significant) are associated in the opposite direction than what would need to be true to explain racial disparities (Black participants score higher than White participants). Thus, concerning race, some beneficial traits are higher in White participants, and others are higher in Black participants. Still, two traits are significantly associated with race in the predicted direction (cognitive ability and trustworthy/self-controlled). Because some trait-theory predictions are supported, we conclude that this element (Table 1, Column 1) is not the main reason that trait explanations fail to account for health inequalities.

### Do Cognitive and/or Noncognitive Traits Explain Inequalities in Self-Rated Health?

Table 2 presents 10 regression equations that (1) document the existence of race and SES inequalities in SRH, (2) assess whether traits are independently associated with SRH as they must be to support a trait explanation for health inequalities, and (3) test whether traits explain health inequalities. Supplemental Tables S11 and S12 in the online version of the article show results for SRH and AL with indicators of SES assessed separately.

**Table 2.** Multiple Regression Equations Assessing Whether Cognitive and/or Noncognitive Traits Account for Race and SES Inequalities in Self-Rated Health: Regression Coefficients (Standard Errors), *n* = 603, Child Health and Development Disparities Study.

	Equation 1	Equation 2	Equation 3	Equation 4	Equation 5	Equation 6	Equation 7	Equation 8	Equation 9	Equation 10
Self-identified Black race	−.546*** (.082)	−.372*** (.092)	−.265** (.089)	−.233* (.093)	−.246** (.090)	−.286** (.090)	−.272** (.089)	−.298*** (.092)	−.248* (.098)	−.243* (.100)
Adult SES			1.531*** (.202)	1.471*** (.209)	1.452*** (.207)	1.485*** (.204)	1.483*** (.204)	1.475*** (.206)	1.345*** (.214)	1.386*** (.216)
Other race-ethnicity	−.374** (.142)	−.278* (.142)	−.379** (.137)	−.365** (.137)	−.385** (.137)	−.375** (.137)	−.370** (.137)	−.372** (.137)	−.363** (.137)	−.361** (.137)
Female	−.060 (.077)	−.053 (.076)	−.053 (.073)	−.041 (.074)	−.063 (.073)	−.064 (.073)	−.051 (.073)	−.037 (.074)	−.059 (.076)	−.072 (.076)
Age	−.051 (.031)	−.048 (.031)	−.049 (.029)	−.058 (.031)	−.056 (.030)	−.049 (.030)	−.048 (.029)	−.052 (.030)	−.061* (.031)	−.055 (.031)
Child SES		.801*** (.203)	.369 (.203)	.304 (.210)	.361 (.202)	.404* (.203)	.362 (.202)	.351 (.203)	.335 (.212)	.290 (.211)
Cognitive ability				.298 (.260)					.219 (.263)	.240 (.266)
Trustworthy/self-controlled					.357 (.204)				.317 (.207)	.321 (.207)
Valued noncognitive traits						.547 (.306)			.435 (.319)	.470 (.325)
Mastery							.422 (.228)		.305 (.275)	.239 (.277)
Self-esteem								.323 (.223)	.014 (.280)	.021 (.276)
Child health										−.521* (.220)
Parents separated/divorced										.021 (.093)
Parental problem with drugs or alcohol										–.216 (.115)
Close to mother as adolescent										.237 (.254)
Constant	6.246*** (1.540)	5.689*** (1.528)	5.147*** (1.463)	5.507*** (1.497)	5.201*** (1.470)	4.692*** (1.491)	4.742*** (1.479)	5.104*** (1.465)	4.914*** (1.536)	4.554** (1.542)

*Note:* SES = socioeconomic status.

* *p* < .05, ** *p* < .01, ***p < .001.

#### Are there independent race and SES inequalities in SRH?

The first three equations in Table 2 show results relevant to the interplay between race and SES as each is associated with SRH. Equation 1, which includes only race-ethnicity, sex, and age, shows that Black participants have substantially lower (*b* = –.546, *p* < .001) SRH than White participants. Equation 2 adds child SES, and then Equation 3 further adds adult SES. Consistent with the possibility that racism harms health by depressing socioeconomic conditions across the life course, the coefficient for Black race declines to *b* = –.265 (*p* < .01) in Equation 3. Consistent with the possibility that racism has untoward health consequences independent of SES, the coefficient for race remains substantial and significant. Regarding SES, Equation 2 shows that childhood SES has an independent association with SRH (*b* = .801, *p* < .001) that is substantially reduced when adult SES is controlled (*b* = .369, n.s.). As Equation 3 shows, adult SES is strongly associated with SRH (*b* = 1.531, *p* < .001), with the predicted difference between the highest and lowest SES score being more than 1.5 points on the 1 (poor) to 5 (excellent) SRH measure.

#### Are trait variables independently associated with SRH (testing Table 1, Column 2)?

Equations 4 through 8 in Table 2 add each of the traits separately to determine whether they are significantly associated with SRH. As Table 2 shows, none of the five trait variables is significantly associated with SRH when age, sex, race-ethnicity, and child and adult SES are controlled. This conclusion is also true in Equation 9, when all traits are entered together, and in Equation 10, when controls for child health and family-context factors are also added.

#### Do trait variables explain race and SES inequalities in SRH (testing Table 1, Column 3)?

Turning to the issue of whether controls for traits explain health inequalities, we examine the coefficients for race and SES to determine whether they become nonsignificant as we enter the traits singly or in unison in Equations 4 to 9 in Table 2. As Table 2 shows, the coefficient representing health inequalities for Black participants remains statistically significant across all equations, as does the coefficient for adult SES, indicating that neither singly nor in combination do traits explain social inequalities in SRH. When traits are entered singly, the largest decline in the proportion of the race effect explained is 12.1%, which occurs when cognitive ability is entered in Equation 4 (*b*s = –.265 to –.233). For adult SES, the largest proportion explained, 6.5% (*b*s = 1.531 to 1.452), occurs when trustworthy/self-controlled is entered. In summary, results align with Table 1, Row 3, because traits are not independently associated with SRH and do not explain health inequalities by race or SES.

### Do Cognitive and/or Noncognitive Traits Explain Inequalities in Allostatic Load by Race and SES?

#### Are there independent race and SES inequalities in AL?

Table 3, Equation 1 shows that Black participants experience more indicators of AL than White participants (*b* = .456, incidence rate ratio = 1.578). Again, in keeping with the possibility that racism harms health by depressing child and adult SES, the coefficient for Black race declines to *b* = .257 in Equation 3 but remains independently and significantly associated with AL. Concerning SES, Equation 2 shows that childhood SES has an independent association with AL (*b* = –.734, incidence rate ratio for top vs. bottom of SES distribution = .480) that is notably but not entirely reduced when adult SES is controlled in Equation 3 (*b* = –.563, incidence rate ratio = .569). Thus, the results presented in Columns 1 to 3 of Table 3 show that, as with adult SRH, inequalities in AL are evident for race and SES.

**Table 3.** Negative Binomial Regression Equations Assessing Whether Cognitive and/or Noncognitive Traits Account for Race and SES Inequalities in Allostatic Load: Regression Coefficients (Standard Errors), *n* = 400, Child Health and Development Disparities Study.

	Equation 1	Equation 2	Equation 3	Equation 4	Equation 5	Equation 6	Equation 7	Equation 8	Equation 9	Equation 10
Self-identified Black race	.456*** (.091)	.312** (.100)	.257* (.102)	.227* (.109)	.245* (.102)	.244* (.102)	.261* (.102)	.274** (.105)	.238* (.113)	.254* (.116)
Adult SES			−.616* (.242)	−.568* (.250)	−.522* (.248)	−.672** (.243)	−.600* (.245)	−.592** (.245)	−.514* (.256)	−.539* (.259)
Self-identified other race-ethnicity	.153 (.160)	.054 (.161)	.086 (.161)	.065 (.163)	.068 (.161)	.097 (.160)	.088 (.161)	.083 (.161)	.065 (.162)	.071 (.163)
Female	−.198* (.087)	−.219* (.086)	−.231** (.086)	−.241** (.087)	−.226** (.086)	−.246** (.086)	−.231** (.086)	−.238** (.086)	−.260** (.088)	−.263** (.089)
Age	−.008 (.034)	−.011 (.034)	−.005 (.034)	.003 (.036)	.002 (.034)	.000 (.033)	−.006 (.037)	−.004 (.034)	.016 (.036)	.017 (.036)
Child SES		−.734** (.233)	−.563* (.241)	−.516* (.249)	−.593* (.241)	−.527* (.241)	−.569* (.241)	−.535* (.242)	−.496* (.254)	−.499* (.257)
Cognitive ability				−.238 (.315)					−.138 (.315)	−.139 (.320)
Trustworthy/self-controlled					−.347 (.220)				−.358 (.224)	−.366 (.226)
Valued noncognitive traits						.650 (.367)			.840* (.396)	.854* (.397)
Mastery							−.111 (.245)		−.101 (.313)	−.064 (.317)
Self-esteem								−.174 (.264)	−.268 (.349)	−.265 (.353)
Child health										.037 (.257)
Parents separated/divorced										−.066 (.105)
Parental problem with drugs or alcohol										.041 (.126)
Close to mother as adolescent										−.217 (.289)
Constant	1.174 (1.691)	1.713 (1.675)	1.671 (1.661)	1.316 (1.731)	1.529 (1.664)	.889 (1.719)	1.777 (1.675)	1.702 (1.662)	.451 (1.796)	.534 (1.811)

*Note:* SES = socioeconomic status.

* *p* < .05, ** *p* < .01, ***p < .001.

#### Are trait variables independently associated with AL (testing Table 1, Column 2)?

Equations 4 through 8 in Table 3 show no instance in which any trait variable is significantly associated with the count of AL when entered one at a time into an equation controlling for age, sex, race-ethnicity, and child and adult SES. Equation 9, which enters all traits together, and Equation 10, which further enters all covariates, show a significant association between valued noncognitive traits and AL, but the direction is opposite to what would need to be true for the trait to explain health inequalities. Participants who score higher on valued noncognitive traits have higher counts of AL.

#### Do trait variables explain race and SES inequalities in AL (testing Table 1, Column 3)?

Equations 4 through 8 in Table 3 enter each trait measure alone, and then Equation 9 enters them all together. Examining the race and SES coefficients across Equations 4 through 9, we see that neither falls to nonsignificance as trait variables are entered individually or in combination. Using PARAMED (see Supplement Section 6 in the online version of the article) to assess the percentage of race and SES associations explained, we find the largest decline occurs when cognitive ability is entered (Equation 4), but the estimated 12.4% accounted for is relatively small and nonsignificant. Turning to inequalities by adult SES, we observe that a statistically significant association is consistently observed across Columns 4 to 9 in Table 3. The largest reduction in the adult SES coefficient is 10.9%, which occurs when the variable trustworthy/self-controlled is entered in Equation 5. In sum, the evidence is consistent with Table 1, Row 3, because traits are not independently associated with AL and do not explain health inequalities by either race or SES.

### Evidence Relevant to the Importance of Comprehensive Life Course Measurement of SES

Although relatively few studies have had the requisite life course data to test trait explanations for health inequalities, two, discussed in the introduction, claimed support for such explanations based on analyses of the British Cohort Studies (Carter et al. 2019; Oi and Alwin 2017; see Supplement Section 2 in the online version of the article). One followed participants only through age 30 and measured adult SES with a single indicator of educational attainment (Oi and Alwin 2017). The other (Carter et al. 2019:196), like ours, followed participants through age 50 and concluded that noncognitive traits were “potentially valuable target for policies aiming to ameliorate the production of health inequalities.” However, the study operationalized what are known to be graded relationships with dichotomous indicators of education and occupation while at the same time excluding measures of income and wealth. To explore the possibility that the comprehensive measurement of adult SES matters, we used the same indicators of SES and dichotomized them in the same way as the Carter et al. (2019) study. We found, as reported in Table S13 in the online version of the article, that had our study measured SES in this truncated, incomplete fashion, we also would have concluded that cognitive and noncognitive traits had independent associations with SRH.

### Evidence for Interaction between Traits and Race and Traits and SES on Health Outcomes

We tested interactions between the five measured traits, the two health-inequality variables (race and adult SES), and the two health outcomes (adult SRH and AL) and found 3 of 20 to be significant. They involved interactions between self-identified race and cognitive ability, trustworthy/self-controlled, and mastery on adult SRH. They show, as other research has for education and income (Assari 2018), that Black people receive fewer returns than White people for equal levels of a potentially beneficial trait. Table S14 in the online version of the article shows equations including these interactions, and Figure S2 in the online version of the article graphs them. The results reveal that at no point within the distribution of any of the three traits did White participants have a worse predicted SRH than Black participants. Additionally, a trait explanation for health inequalities might be considered an equalizer if the gap between Black and White participants was smaller when cognitive ability, trustworthy/self-controlled, or mastery was higher rather than lower. However, the results are just the opposite for all three traits: The racial gaps are larger when trait levels are higher. Although we suggest caution in interpreting these interactions because only 3 of 20 emerged, still to the extent that they do confer evidence, they do not support trait explanations for racial inequalities in health.

### Evidence Relevant to the Consistency of Conclusions When Complete Case Analysis Is Used

As a sensitivity check, Tables S15 and S16 in the online version of the article show complete-case analyses that involved no imputation at all. In 10 tests (5 traits × 2 health outcomes), one of the trait variables (trustworthy dependable) showed a barely significant (*p* = .04) independent effect on SRH (but not AL). All of the other inferences about traits were the same in the complete-case and multiple-imputation analyses, and most importantly, in no case are health inequalities by either race or SES explained by the entry of trait variables either singly or in combination.

## Discussion

### The Issue

Strong claims that cognitive and noncognitive traits are implicated in the generation of health inequalities by race and SES have been prominently proffered in the literature. Developed early in life, these traits, the argument goes, are important reasons why health inequalities by race and SES are so regularly observed. Addressing health (and other) inequalities requires intervening early in the life course of people from disadvantaged groups to overcome what is claimed to be a deficient parental investment in children to help youth develop beneficial traits that will lead them to a prosperous and healthy life. Given the strong correspondence between trait explanations and the American Dream notion that it is in the exercise of such traits that one’s fate in American society is secured, we deemed it important to turn a sociological eye to whether trait explanations play a substantial role in explaining health inequalities by race and socioeconomic status.

### The Evidence Concerning Traits as Explanations for Health Inequalities

We found no strong evidence that cognitive or noncognitive traits explain racial or SES-based health inequalities when SES is comprehensively assessed. Across both outcomes (SRH, AL), the maximum decrease in the magnitude of race or SES coefficients with the addition of any trait variable was 12.4%, and in no instance did a race or SES coefficient move to nonsignificance when traits were controlled. Instead, the results showed that it was controls for race and SES that accounted for any bivariate association between traits and health outcomes. In no instance was any trait measure significantly independently associated in the expected direction with either health outcome when race and SES were controlled. These results are incompatible with the possibility that cognitive and noncognitive traits play a substantial role in explaining health inequalities.

### Considering the Possibility of Indirect Effects of Traits on Health

Given our finding that some of the traits we assessed were independently associated with adult socioeconomic attainment, results are consistent with the possibility that cognitive and noncognitive traits affect SES, which then affects health. In such a scenario, traits are potentially helpful for moving up the socioeconomic ladder to yield the multiple benefits that a higher SES location confers for many outcomes, including health. Although our main research question involves direct effects, because these must be observed for traits to play a substantial role in explaining health inequalities, we examined indirect effects and found them to be significant concerning SRH for all five of the trait variables but only cognitive ability for AL (see Section 7 in the online version of the article). The magnitude of these indirect effects is relatively modest. Concerning the largest indirect effect, a 50% difference in percentile ranking on cognitive ability is associated with a .20-point change on the 5-point SRH scale through adult SES. In comparison, a 50% difference in ranking on adult SES is more than 3 times as large (.74). Nevertheless, because our results provide some evidence for indirect effects of traits through adult SES, further research focused on these indirect pathways has potential value.

However, it is important to recognize that the indirect-effect-only set of findings that we produced strenuously limits claims of cognitive and noncognitive explanations for health inequalities. First, an indirect-effect-only pattern of results for SES is inconsistent with the claim that traits explain large proportions of health inequalities by race and SES. Second, indirect-effect-only findings must assign considerable importance to adult SES because any effect of traits needs to work through SES in affecting health. Finally, mechanistic explanations used to support trait explanations for health inequalities are challenged by an indirect-only pattern of results. For trait explanations to effectively account for health inequalities, we would expect health-beneficial traits to operate effectively within levels of SES and within Black people and White people, but this is not something that we observe.

### Consideration of Limitations and Strengths

As would be expected in an approximately 50-year follow-up study, we were unable to recruit every eligible subject we sought to locate. Nevertheless, we found little evidence of bias either in comparisons based on archived data of recruited and nonrecruited subjects or in comparisons between our sample and the nationally representative NHANES data.

Our data include only individuals who resided in the Bay Area in California when they were initially recruited into the study and thus is limited in generalizability in a way that a nationally representative study would not be. Moreover, the sample represents the populations who were living in that area at the time and, therefore, does not include large enough samples of Latino or other ethnic-racial minority groups for analysis.

Our health measures are purposefully broad, including SRH, which could be less than good or excellent for many reasons, and AL, which assesses biomarkers for multiple body systems, not just one. Although this is appropriate given the issue we engage, it is important to recognize that we have not tested more specific disease conditions and that different results might be obtained in particular cases. With this caveat about particular health conditions in mind, we stand with the appropriateness of the broad measures we employed for the issues that we addressed.

Finally, our analysis does not end up explaining health inequalities—inequalities by race and SES endure in all of our results. In light of this, our study’s contribution is limited to evaluating trait explanations for health inequalities. However, because such explanations have been proposed in the literature, align with the American public’s tendency to emphasize personal “choices” over social conditions in matters of health, and are likely to gain renewed traction in the current political climate, we believe our assessment holds meaningful value.

### Implications for Research and Policy

Our results suggest that prominent, strongly stated claims about the centrality of traits in producing health inequalities need to be tempered and any resulting policy claims or actions questioned. Although specific policies addressing health inequalities should follow from the broader sociological and social epidemiological literatures, our findings on the salience of SES in childhood and adulthood support the potential benefits of improving socioeconomic conditions across the life course—such as access to free or affordable childcare, fair wages, effective health care, and good, equal schools, to name just a few.

Our results also have implications for an issue raised by Link and García (2021) concerning the prominence of what they call “diversions.” Diversions occur when explanations for health inequalities systematically direct attention to the conditions and characteristics of disadvantaged groups and away from the actions of more powerful ones. Trait explanations do this. The fact that we found little evidence supporting trait explanations for health inequalities diminishes the capacity of such explanations to offer a compelling counternarrative to investigations focused on race- and class-based bias and discrimination.

## Conclusion

Theory and evidence from multiple research programs in sociology and social epidemiology point to stress across the life course, fundamental causes, and structural racism to explain health inequalities by race and SES. The trait explanations that we considered direct attention elsewhere, offering a radically different account of the emergence of health inequalities in the United States and globally. And of course, such a trait-based alternative implies a different set of needed policies and interventions. Given this sharp divide in understanding and proposed action, evidence regarding the ability of trait explanations to account for health inequalities carries substantial weight. The prospective data structure that we employed allowed trait explanations a strong opportunity to show that they play a substantial role in explaining health inequalities—something that should have occurred if they were as powerful as their proponents claim. That they did not prompts us to question claims about their capacity to explain why health inequalities by race and SES remain such prominent social facts.

## Supplemental Material

sj-docx-1-hsb-10.1177_00221465251368347 – Supplemental material for American Dreaming? Evaluating Trait Explanations for Health Inequalities by Race and Socioeconomic Status Supplemental material, sj-docx-1-hsb-10.1177_00221465251368347 for American Dreaming? Evaluating Trait Explanations for Health Inequalities by Race and Socioeconomic Status by Bruce G. Link, Ezra S. Susser, Pam Factor-Litvak and Barbara A Cohn in Journal of Health and Social Behavior
