# Supplementary material for: American Dreaming? Evaluating Trait Explanations for Health Inequalities by Race and Socioeconomic Status
Source: J Health Soc Behav. 2025 Oct 23;67(3):487–505. doi: 10.1177/00221465251368347 (PMC13542383; doi:10.1177/00221465251368347)
Supplement: Supplementary file 1 [file sj-docx-1-hsb-10.1177_00221465251368347.docx]

**Journal** of **Health**

and **Social Behavior**

OFFICIAL JOURNAL OF THE AMERICAN SOCIOLOGICAL ASSOCIATION

**ONLINE SUPPLEMENT**

**to article in**

Journal of Health and Social Behavior

**American Dreaming?**

**Evaluating Trait Explanations for Health Inequalities by Race and Socioeconomic Status**

**Bruce G. Link**

*University of California Riverside*

**Ezra S. Susser**

*Columbia University and New York State Psychiatric Institute*

**Pam Factor-Litvak**

*Columbia University*

**Barbara A Cohn**

*Child Health and Development Studies, Public Health Institute*

This online supplement provides additional information about 1) the Abecedarian and Perry studies, 2) the measurement of SES in British Cohort studies, 3) the origin and development of the Child Health and Development Studies (CHDS) and the CHDS-Disparities Study, 4) evidence about sample quality of the present study, 5) additional detail on measurement of allostatic load, race/ethnicity, sex at birth, childhood SES, adult SES, cognition and the index of child health problems, 6) additional information about the use of PARAMED (Emsley and Liu, 2013) to assess whether traits explain race and SES associations with allostatic load, and 7) multiple supplementary tables and figures that pertain to issues raised in the manuscript, that could not be included due to space considerations. Topics are presented in the order they arise in the manuscript.

**#1) Evidence from the Abecedarian and Perry Studies concerning Non-Cognitive Traits**

As the text indicates, a cornerstone of the non-cognitive approach directs attention to two experimental studies. The two studies in question, the Abecedarian and Perry studies, were early-life interventions conducted among mostly Black participants that involved long-term follow-up into adult life (Campbell et al. 2014; Heckman, Pinto, and Savelyev, 2013). The studies are important because the school-readiness interventions they involved are expected, according to a non-cognitive trait explanation, to improve such traits, which would then lead to long-term benefits for adult outcomes including health. While small, the studies show that early life interventions can have lasting, plausibly causal, effects on adult outcomes including health. The critical question for understanding the role of non-cognitive traits, however, is whether any health benefit following the interventions was achieved by changing non-cognitive traits, a finding that would help identify the specific importance of such traits. Only the Perry study included traits, with the ones assessed being "externalizing behaviors" (lies, aggression) and "academic motivation" (shows initiative, alert and interested). Importantly, the intervention only affected one of the traits in both boys and girls (externalizing behaviors), and mediation (intervention → traits → health) was only observed concerning one of the traits (externalizing behaviors) for one of the two health-related outcomes assessed (smoking) and then only among men. Thus, as important as these studies have been considered for demonstrating the potential value of early intervention when it comes to providing support for a non-cognitive explanation for health inequalities, the evidence is not strong (Perry study) or not available (Abecedarian).

**#2) Evidence concerning the measurement of SES in the British Cohort Studies**

As indicated in the text, two studies employing the British Cohorts provided long-term follow-up of prospectively collected non-cognitive traits in relation to SES. Oi and Alwin (2017) use the 1958 cohort and Carter, et al. ( 2019) the 1948 cohort. The Oi and Alwin study concludes that a sense of control (the non-cognitive trait they assessed) is “an important health-related factor originating early in life, influencing both health and education later in adulthood” (p. 198), whereas the Carter et al, (2019) study concludes that because traits show independent associations with health, they “could be a target for policies aimed at ameliorating the production of health inequalities” (Carter et al. 2019, p. 190). As indicated in the text, both studies are based on homogeneously White populations, making it impossible to investigate racial health inequalities, are restricted to self-report outcomes, and with respect to the Oi and Alwin study only follow participants only through age 30. However, the most consequential issues involve the life course measurement of SES. The Oi and Alwin study assessed multiple aspects of SES up to age five (but not after 5) but only educational attainment at age 30. The Carter et al. (2019) study used a single binary indicator of child SES (father’s manual versus non-manual occupation) and then only binary indicators of educational and occupational attainment in adult life. The dichotomization of what are known to be finely graded associations between SES and health along with the limited coverage of important domains of SES (income and wealth) suggests the possibility that SES may have been inadequately assessed in these studies’ assessments of the independent roles of cognitive and non-cognitive traits.

**#3) Description of the Child Health and Development Studies**

The Child Health and Development Studies (CHDS) began in 1959 as a collaborative effort between the University of California Berkeley School of Public Health, the Kaiser Foundation Research Institute, and the Kaiser Permanente Medical Group with funding from the National Institutes of Health. Participants in the CHDS-Disparities Study are adult children from families that were originally recruited as part of the CHDS. As described in the text, almost all pregnant women receiving prenatal care from the Kaiser Foundation Health Plan at its facilities in Alameda County, California were recruited to the CHDS. The 19,044 live births (1959 -1967) were automatically enrolled in the Health Plan – one of the earliest and still one of the largest pre-paid health plans in the United States. It provided health care to approximately 30% of the population of the county, which includes the cities of Oakland, Berkeley, and Hayward. A later comparison with U.S. census and survey data demonstrated that the Kaiser members were demographically similar to the population of the region (Krieger 1992), though the distribution of SES may be somewhat truncated at each end since Kaiser members were largely employed. Kaiser membership, and by extension the CHDS, which encompassed over 95% of the Kaiser obstetric patient population, was racially, educationally, and occupationally diverse. The CHDS was designed to investigate events of pregnancy, labor, and delivery and to relate these observations to subsequent childhood development (van den Berg, Christianson & Oechsli 1988) but as the cohort has aged, the scope of the inquiry has turned to issues such as the early determinants of schizophrenia, breast cancer, and to issues of human reproduction.

The cohort has been regularly monitored by linkage to: 1) the California Department of Motor Vehicles, for a history of location and timing of residence to identify the population at risk for morbidity and mortality, 2) the California Department of Vital Statistics, for identifying deaths and cause of death (Mongraw-Chaffin, Cirillo & Cohn 2010; Wang et al 2011; Cirillo and Cohn 2015), and 3) the California Cancer Registry, for identifying cancer diagnoses (Cohn et al. 2001; Cohn et al. 2007; Cohn, Cirillo, and Christianson 2010). All members of CHDS families and all names ever used by each family member are regularly matched to these sources. The accumulation of a name and address history for each cohort member protects against establishing false matches and failing to identify true matches. Surveillance efforts routinely identify one or more members of over 80% of CHDS families. Life-table analysis estimating expected numbers of breast and testicular cancer cases shows close comparability to observed numbers (Cohn et al. 2007; Mongraw-Chaffin et al. 2009). Linkages to the National Death Index to evaluate the completeness of mortality surveillance have yielded very few missed cases. These quality control efforts affirm that surveillance of the cohort provides reliable and comprehensive coverage.

The CHDS is well suited for the study of health disparities because it includes sufficient racial and socioeconomic diversity to facilitate a study of such disparities. This diversity resulted from large-scale migration to the East Bay Area from varied locations within the United States which began in the 19^th^ century and accelerated rapidly in the 1930s and 1940s (Moore 2000; Self 2003; Rhomberg 2004). Because eligibility for the CHDS was based on enrollment in the Kaiser Health Plan, some truncation at the *lowest* end of the SES continuum is likely. Still, substantial variations by indicators of SES are present in the data, likely due to the broad range of occupations included in Kaiser membership via unions and contracts (Kaiser Permanente 2016). Comparing the U.S. census with survey data demonstrated that the Kaiser membership produced relatively small differences in exposure to high-poverty census tracts (8.7% health plan members, 13.2% general population) (Krieger 1992).

Recruitment into the age 5, 9-11, and 15-17 follow-up studies is briefly described in the text. Recruitment of 8,343 participants into the age-5 assessment is described in a flow chart provided by van den Berg, Christianson, and, Oechsli (1988). Following the age-5 recruitment, several factors influenced the development of the pool eligible for our study. One important one was that each subsequent wave of childhood assessments required participation in the prior waves. Another was that because of the costs of in-person interviews, recruitment for psychological testing and the collection of biological data was limited to families who remained in the Bay area. Loss to follow-up for this reason existed but was not especially large in this relatively stable population. Further, the 9-11 recruitment was attempted only from mothers with children born between April 1960 and April 1963 thereby limiting, for our purposes, the cohort to youth from the original cohort who were born within that window. Finally, despite extensive efforts to trace the cohort, loss to follow-up occurred leading us to efforts to assess the validity of the sample by comparing our recruited sample to those not recruited on data from the baseline assessment. In the end, as stated in the text, a pool of 3,196 eligible participants was identified.

As described in the text, the study attempted to contact 1,633 of the identified pool of 3,196 eligible CHDS sons and daughters who had participated in a childhood follow-up study and who were not deceased, not known to have moved out of California and not known to be ineligible for other reasons (e.g. current incarceration). This online supplement provides additional details about each step in the recruitment. As Figure 1 shows, we were unable to find a current address for 111 potential participants (7%). The remaining 1,522 were contacted by mail with a letter from the investigators introducing the study and offering potential participants the opportunity to participate or refuse participation. Potential participants were also asked to provide a telephone number and good times to reach them by telephone. To boost responses to this letter we included a $5 bill as an incentive to participate. Telephone numbers were ascertained by response to the mailed invitation and by information previously collected from cohort members between 2005 and 2009, requests for contact updates from family members, and online searching. We were able to obtain telephone numbers for 1073 (71%). We then proceeded to contact people by telephone and were able to achieve the desired targets for each race and gender group from 985 of the 1073 (13 were never attempted and 75 were in the process of recruitment but truncated when target numbers were achieved). Telephone contacts were attempted by the call center staff of Examination Management Services Incorporated (EMSI) either at the times that participants indicated might be best for them or at varied hours and days of the week including evenings and weekends. Multiple calls (at least 10) were attempted before considering a potential participant to be a passive refusal. We conducted a computer-assisted telephone interview (CATI) with 605 (61%) of the 985 individuals we attempted to interview. Of the 378 we did not interview, 238 (24.5% of the 985) could not be reached even after multiple attempts and were thereby considered passive refusals; the remaining 142 (14.5% of the 985) actively refused participation. Once respondents agreed to the telephone interview, we also sought their participation in the home visit and self-administered questionnaire components of our study. As Figure 1 shows, of the 605 individuals who participated in the telephone interview, 510 (84%) completed the home visit and 497 (82%) completed the self-administered questionnaire. Concerning race, of the 605 participants, 310 (51.2%) were classified as non-Hispanic white, 244 (40.3%) as Black, 17 (2.8%) as Latino, 23 (3.8%) as Asian, and 11 (1.8%) as other.

Figure S1 About Here

As indicated in the text Table S1 provides details about the participation of participants in the waves of data collection conducted when the participants were children. A majority of participants (69%, N=420) were involved in all four child assessments, some in three (16.2%, N=98), and only a small proportion (14.2%, N=87) were involved in only 2. None were involved in only one.

Table S1 About Here

**#4) Evidence Pertaining to Sample Quality of the CHDS-Disparities Study**

Comparison of Recruited to non-Recruited on Characteristics from Archived Data. We assessed the possibility that those who responded may have differed from people who did not on characteristics ascertained in earlier waves of CHDS data collection. Results are presented separately by race because we over-sampled African Americans (as described in the text). To achieve our sampling target for African Americans we extended recruitment to numerous subjects who had not participated in the adolescent CHDS study but had participated in the 9-11 and/or the 5-year-old assessments. Because of over-sampling and differential recruitment methods by race, the best and most complete representation of predictors of non-response is to report results separately by race. We did not seek to recruit potential participants who moved out of California (21% did). As a result, we examined whether movers differed from stayers on mother’s age, education, employment, father’s age, education and employment, family income, and offspring birthweight. Neither for White nor Black participants did we find any significant differences (p<.05) between those remaining in California and those who left (data not shown). Only a small number of cohort members had died (8 White and 8 Black people). Restricting to individuals alive and living in California, Table S2 compares contacted cohort members who participated to those who did not. As the table shows, there are no significant differences for any of the variables assessed.

Table S2 About Here

Comparison of the CHDS-Disparities Study to NHANES. As described in the text, we wanted evidence regarding the representativeness of our California-based sample and as a consequence undertook a comparison with the NHANES (National Health and Nutrition Examination Survey 2016) as a nationally representative reference that contained some of the variables we measured, including biomarkers. While the cross-sectional NHANES has the advantage of representativeness, it does not have the prospectively collected life-course assessment of SES and cognitive and non-cognitive traits. Tables S3 and S4 answer three questions: 1) are the samples different with respect to the indicators assessed? 2) are there race and education disparities evident in each sample? and 3) does the magnitude of any disparity significantly differ across the two samples? With respect to the first question, we find only modest differences between the samples; BMI is slightly lower among women (30.5 versus 32.1) in the CHDS-Disparities Study and self-rated health slightly better, with 82% rating their health as good, very good or excellent in our sample versus 78% for the same categories in NHANES. With respect to the second question, substantial and expected disparities by both race and education are generally evident and comparable in both samples. One exception is that among men in the CHDS-Disparities Study, African Americans have a significantly higher BMI than Caucasians, a disparity that is not statistically significant among men in NHANES. Education disparities are somewhat less consistent but generally show the same pattern between the two samples. With respect to our third question, in no instance was a race or education disparity significantly different between the samples. This latter finding is particularly important because it indicates that the disparities we seek to explain in the disparities sample are not appreciably different from the disparities that exist in the nationally representative NHANES.

While the NHANES provides an important point of comparison that helps ensure that our regional data contain roughly the same disparities as nationwide data, it is important to observe that NHANES data cannot convincingly achieve an understanding of the developmental life-course factors that lead to the observed disparities. The only way it could seek to do so would be to collect life-course data retrospectively. While this can be useful for some life-course variables, knowledge or memories about things like family income at birth, blood pressure at age 15, BMI at age 15-17, or critically the cognitive and non-cognitive traits that are the focus of this paper could not be reliably assessed at age 50. Factors like the ones we assessed prospectively could help explain why disparities in adult life exist. A contribution of our study that could not be achieved with NHANES is to evaluate which of the many factors we prospectively measured are in fact important for adult disparities.

Table S3 and S4 About Here

**#5) Additional detail on the measurement of sex/gender, race/ethnicity, adult SES, childhood SES, allostatic load, cognitive ability, and the index of child health problems.**

**Allostatic Load**. The 10 indicators used to assess allostatic load were ascertained in the home visit and included measurement of height and weight to obtain body mass index (BMI), waist circumference (WC) (Indicator 1), percent body fat which we use instead of BMI (%BF) (Indicator 2) and systolic and diastolic blood pressure (BP) (Indicators 3 and 4), collection of information on medication use, and a blood draw. The blood draw, along with the information on medication use, provides the final six indicators: hemoglobin A1C (HbA1c) (Indicator 5), high-density lipoprotein (HDL) cholesterol (Indicator 6), ratio total/HDL cholesterol (Indicator 7), dehydroepiandrosterone sulfate (DHEAS) (Indicator 8), interleukin-6 (IL-6) (Indicator 9), and C-reactive protein (CRP) (Indicator 10). A licensed phlebotomist attended each home visit, and home-visit personnel were trained in all study measurements to facilitate reliable measurement. The method for obtaining each indicator is described below based on an in-depth description of study procedures by Kezios et al. (2022). Participant height (cm) was measured using a seca 217 stadiometer (seca GmbH & Co. KG., Hamburg, Germany), while weight (kg) and %BF were measured using a TANITA BF-350 Body Composition Analyzer (TANITA Corporation, Tokyo, Japan). A FitnessMart Gulick II Tape Measure (Model 67020, Country Technology, Inc., Wisconsin, USA) was used to obtain WC (cm). Each measure was taken twice and the average of the two measures was used for analysis.

Systolic BP (SBP) and diastolic BP (DBP) measurements were assessed three times using automated monitors (CareScape V100 Vital Signs Monitor, GE Healthcare, Wisconsin, USA) and, as is standard procedure, the average of the last two measurements was used to calculate mean BP values.

In total, 20 mL of serum and 40 mL of whole blood were collected, processed within two hours, and shipped on dry ice for storage in the laboratory of Dr. Regina Santella at Columbia University's Center for Environmental Health in Northern Manhattan. Serum samples were then shipped to the Clinical & Epidemiologic Research Laboratory at the Boston Children's Hospital for analysis of hemoglobin A1C (HbA1c), total cholesterol, high-density lipoprotein (HDL) cholesterol, dehydroepiandrosterone sulfate (DHEAS), interleukin-6 (IL-6), and C-reactive protein (CRP). Following best practices, samples were batched in a random order for analysis.

Total cholesterol was measured using standard methods. At cholesterol concentrations of 132.8 and 280.4 mg/dL, the day-to-day reproducibility, reflected by the coefficient of variation (CV), was 1.7% and 1.6%, respectively. HDL cholesterol concentrations were determined using a direct enzymatic colorimetric assay (Sugiuchi et al., 1995). This assay has been shown to meet the requirements established by the Lipid Standardization Program (Rifai et al., 1998). At HDL concentrations of 27.0 and 54.9 mg/dL, the day-to-day reproducibility was 3.3% and 1.7%, respectively.

CRP concentrations were determined using an immunoturbidimetric assay on the Roche P Modular system (Roche Diagnostics – Indianapolis, IN), using reagents and calibrators from Roche. This high-sensitivity assay has a limit of detection of 0.03 mg/L. The day-to-day variabilities of the assay at concentrations of 0.91, 1.60, and 18.40 mg/L were 3.8%, 3.3%, and 1.9%, respectively.

DHEAS was measured by a competitive electrochemiluminescence immunoassay on the Roche E Modular system (Roche Diagnostics, Indianapolis, IN). The lowest detection limit of this assay is 0.1 μg/dL and the run-to-run imprecision values at DHEAS concentrations of 117, 395 and 984 μg/dL were 2.8%, 2.4%, and 1.7%, respectively.

Determination of the %HbA1c in total Hemoglobin was based on turbidimetric immunoinhibition using hemolyzed whole blood or packed red cells (Roche Diagnostics, Indianapolis, IN). The assay is certified by the National Glycohemoglobin Standardization Program for no interference by Hb C trait, Hb S trait, Hb E trait, elevated HbF, or Carbamyl-Hb. HbA1c measurements of blood from long-term storage are highly correlated to measurements from blood obtained before storage (Selvin et al., 2005). The day-to-day variability at %HbA1c values of 5.5 and 9.1 are 1.9% and 3.0%, respectively.

IL-6 was measured by an ultra-sensitive ELISA assay from R & D Systems, Minneapolis, MN. The minimum required volume for this assay is 200 μL. The assay has a sensitivity of 0.094 pg/mL, and the day-to-day variabilities of the assay at concentrations of 0.49, 2.78, and 5.65 pg/mL were 9.6%, 7.2%, and 6.5%, respectively.

At the home visit, examiners completed a medication inventory form by documenting the name, dose, and frequency of every medication (prescription and non-prescription, such as supplements) a participant was taking, with information obtained from medication bottles provided by the participant rather than the participant’s self-report. Based on the medications participants in our study were taking, we created categories for medications used to control blood pressure (e.g., ACE inhibitors), cholesterol (e.g., statins), and diabetes (e.g., insulin and oral medications). For components where a participant did not exceed the biomarker high-risk quartile but was taking medications, scores were recoded to 1 to capture underlying subsystem dysregulation masked/controlled via medical intervention (91, 40, and 19 participants’ scores were recoded for SBP, HDL, and HbA1c, respectively).

**Race/Ethnicity**. As indicated in the text, all classifications are based on the self-identification of study participants with 310 (51.2%) identifying as white, 244 (40.3%) as Black, and 51 (8.4%) as some other race/ethnicity. There is a strong correspondence between participants’ self-identifications of Black versus White race and the pre-recruitment classification of race based on parental race. Only one participant was discrepant, self-identifying as White when the study had classified him as Black based on his mother's and father's race. The social circumstances shaping the Bay Area of California when the study began in the late 1950s resulted in a population that consisted of mainly Black and White people. As a consequence, other groups were present only in smaller numbers (N=51). Of these 51, 21 identified as Asian, 15 as Hispanic, 1 as American Indian, and 15 as something else.

**Sex/Gender**. As this was a long-term follow-up study of people who had previously participated in the Child Health and Development Studies we had records of their sex as assigned at birth. In our telephone interview, participants were approached as follows: “It says here that you are [male/female], is this correct? In no instance did a respondent provide a response that disagreed with the record. We decided the best way to characterize what we assessed is “sex assigned at birth.” We indicate this in the first mention of the categorization of participants on this variable in the text and then use the term “sex” in the remainder of the paper.

**Childhood SES**. Family income during childhood was assessed by the self-report of mothers. Because income data were collected in slightly different ways at the different waves of data collection, we constructed standardized scores. A drawback of standardized scores is that they are sample-specific and in our case time-period specific and cannot, for example, be used to answer questions about rising or declining levels of absolute income over time. But the scores do give a useful assessment of where individuals stand relative to others at the same time point. Only 13 subjects in our study are missing on all three income measures. For missing values, we impute standardized income using multiple imputation as described in the text. Then as described in the text, we averaged the three standardized scores, assigned the lowest such score 0, and the highest 1 with all other values arrayed proportionally based on their standard scores between these two endpoints. We use a three-category variable of father's occupation to operationalize this component of SES with professional, technical, and managerial as the highest category (3); sales and skilled labor as the middle category (2); and service and laborer as the lowest category (1). We then added these measures across the data points assigning the top score a 1 and the bottom score a 0 with all other scores arrayed continuously in between. Because in this report we are interested in the general socioeconomic circumstances of the offspring, we combine maternal education, paternal occupation, and family income into a single variable, weighting each equally by adding each of the three indicators and dividing by 3 to provide a continuous measure of SES circumstances during childhood. The disadvantages of this approach are that the effects of SES at different points in childhood are left unexamined, and differential effects of the components of SES are not investigated. The advantage of the approach is that it provides a single robust assessment of childhood SES using as much information as possible to capture it. And, of course, future investigations can use the separate indicators at separate time points when questions pertinent to that level of detail are paramount.

**Adult SES.** As described in the text we use a composite measure that combines years of education, income, occupational standing, and wealth. Our reading of the literature is that some investigators prefer to measure aspects of SES separately while others prefer a composite. We recall that one of the earliest approaches -- Hollingshead and Redlich – created a composite to operationalize class/SES. Later some in the structural equations model tradition explicitly conceptualized SES as a latent variable considering whether it was "reflective" (arrows from the latent variable) or "formative" (arrows to the latent variable), or something else (Bollen and Bauldry 2011). Status attainment literature, as one example, separated the different dimensions to capture the flow from education to occupational standing to income (etc.). To gauge more recent use, we examined the 2024 issue of JHSB. We found that investigators continue to use both approaches and that in our reading they chose the approach that made the most sense given the empirical question they were investigating. If the investigator held a specific interest in one aspect (or separate aspects) of SES they measured it separately, if they were interested in what was common to or shared by different aspects they used a composite. Two papers one by Smith Greenway et al. (2024) and another by Luo and Wei (2024) are interesting because they used both approaches in each of their papers. Each paper was interested specifically in the effect of education and so measured it separately but also used a composite to capture and control other aspects of SES. With traditions behind each approach and with the current medical sociology literature using both we think the key issue is the rationale for using one versus the other.

While a case can be made for either one, we have chosen to highlight a composite measure but also implement a supplementary analysis (see Tables S13 and S14) that separates the dimensions of adult SES. There are three main reasons for our decision. First, the issue we seek to address does not present a prominent or well-motivated reason for being interested in the unique effect of any one dimension of SES separate from the others. If it did, then separating them would certainly make sense. Second, we are interested in what is shared by (as opposed to unique to) the different dimensions. Put another way we do not want to hold constant/remove from consideration the variance that is shared by, for example, income and education. Our theory test is not only about what is unique to each dimension but what is common to them. We think this is best captured in a composite. Third, and somewhat less important than the other two is that considering each dimension separately produces a much more complex analysis. If we implemented such an approach for childhood and adult SES there would be seven SES variables instead of two. Certainly, if we had a focused interest on each dimension separately this would be valuable but since we do not, we are inclined to the composite approach.

We further adopt a relative measure that ranks people from a high of 1 to a low of 0 versus an absolute measure that captures, for example, actual dollar amounts for income/wealth (e.g. 40, 70, 100K, etc.) and years of education (8, 12, 16, etc.). There is debate about which approach to use which is covered in a paper by Brady and colleagues (Brady et al 2023). Two issues led us to adopt the relative approach. First, because we sought a composite measure (see above), we reasoned that the summing of ranks across the dimensions of education, income occupational standing, and wealth made some intuitive sense – get the average ranking of people on these dimensions. Second, the paper by Brady and colleagues (2023) compared relative and absolute income in the Panel Study of Income Dynamics and found that the relative measure was more strongly related across time to health outcomes. But neither of these considerations yields a "written in stone" rationale for relative measures and as such we have also implemented an approach that operationalizes the measures in absolute terms. We report these results in Table S12 and S13 below.

**Measures of Cognitive Ability.** The Peabody picture vocabulary tests (PPVT) were administered to participants at 9-11 and 15-17 waves of data collection and the Raven progressive matrices were administered at 9-11. The PPVT involves presenting recipients with multiple pages with four pictures each. Then an examiner describes one of the pictures in each set and asks the recipient to identify it. It was developed as a relatively short 20 - 30 minute test to measure verbal intelligence (Dunn and Dunn 1959). The Raven progressive matrices consist of a visual design with a missing piece. The test taker is asked to choose from several choices to complete the missing piece. The Raven has been widely used and is considered a measure of general human intelligence (Raven 1989). The bivariate correlations between the cognitive ability measures are quite high the PVVT at 9-11 is correlated .70 with the PPVT at 15-17 and .55 with the Raven at 9-11. The Raven at 9-11 is correlated .52 with the PPVT at 15-17. We decided to measure cognitive ability as a composite measure because our interest was not in the unique -- partialled for the other measures – effect of any of the cognitive ability tests. We did not think it would be a fair test of the cognitive ability explanation to demand that a particular measure have a unique effect on adult health. Separating the cognitive ability measures in the analysis leads to no changes in our main conclusions. None of the separate cognitive ability measures are significantly associated with health, a joint test of the three separate cognitive measures is non-significant, and neither race nor SES health inequalities are explained with the three cognitive measures entered separately.

**Measure of Trustworthy Dependable.**

The measure trustworthy/self-controlled that is in question comes from the mother's ratings at 9 years old. The items in it closely map onto well-established measures of "externalizing symptoms." We compared the 20 items in our measure to symptoms from the Achenbach Child Behavior Checklist which is perhaps the most widely used measure of child psychiatric symptoms (Achenbach et al. 2016). As shown below the items in our measure have direct or very close analogs in the Achenbach indicating a close overlap with what psychiatric epidemiologists call "externalizing symptoms."

| Item in Trustworthy/Self-controlled Scale | Analog in Achenbach Child Behavior Checklist |
| --- | --- |
| Truthful – does not tell lies. | Lying or cheating |
| Keeps his or her word | Lying or cheating  Breaks rules at home, school, or elsewhere |
| Takes good care of his/her possession | Destroys his/her own things |
| Steals | Steals at home  Steals outside the home |
| Daredevil | Impulsive or acts without thinking |
| Flares up easily | Screams a lot |
| Temper explosions | Temper tantrums or hot temper |
| Jealous of other children | Easily jealous |
| Has to win | Bragging boasting |
| Fights to get own way | Gets in many fights  Physically attacks people  Threatens people |
| Minds without a fuss | Disobedient at home  Disobedient at school |
| Argues | Argues a lot |
| Stubborn | Stubborn, sullen, or irritable |
| Bosses other around |  |
| Sassy talks back | Argues a lot. |
| Hates to sit still | Can’t sit still restless |
| Likes attention | Demands a lot of attention |
| Bullies others | Cruelty, bullying, or meanness to others |
| Starts fights | Gets in many fights |
| Likes to tease | Teases a lot |

As mentioned in the text the non-cognitive literature has constructed a broad tent that has included measures that are referred to using other names in other literatures. Examples are mastery, self-esteem, rotter internal-external locus of control, as well as what psychiatric epidemiologists call “externalizing symptoms.” Heckman and colleagues (Heckman Pinto, and Savelyev 2013) use such an “externalizing” measure to explore potential non-cognitive mechanisms linking experimental results from the Perry study of enriched childhood education to adult outcomes. If we think from the vantage point of the non-cognitive trait perspective, we agree with Heckman et al.’s choice of an externalizing behaviors/symptoms measure and have followed them and others in using such a measure to capture an element of non-cognitive traits. The idea, as we see it, is that having many negative behaviors (lying, cheating, argumentative, impulsive) is arguably debilitating with regard to social advancement. Having fewer of them is less so. Fortunately, our measure also includes positive behaviors (truthful, dependable, takes good care of possession) helping to create a trait continuum from trustworthy/self-controlled to lacking these attributes. For all these reasons we think the measure fits the non-cognitive argument well.

**Child Health Index** includes the following indicators: 1) gestational age 258 days or less, 2) one or more severe anomalies by age 5 (with severe defined as a threat to development or well-being),3) one or more hospitalizations by age 5, 4) heart problem identified at age 5 physical exam, 5) other health-related deviation identified at age 5 physical exam, 6) measured systolic blood pressure at 5-year exam greater than 95th percentile of norms developed from the Pediatric Task Force database and based on age, gender, and height, 7) mother report at age 15–17 of child ever being diagnosed with high blood pressure or measured blood pressure above 95% of CDC norms, 8) mother's report at age 15–17 of child ever being diagnosed with asthma, 9) hospitalization during year before 15–17 interview. Each indicator is assigned a "1" for present and a "0" for absent. Our approach to constructing an index of child health problems has the benefit of allowing an efficient gauging of general health across many indicators. Each indicator taken alone applies to relatively few people and therefore confers relatively little power for assessing outcomes that might be related to child health. On the other hand, creating such an index brings two problems. First, if the research question involves a very specific outcome and if one of the indicators has a strong connection to that outcome, adding other indicators to the index could cloud the capacity to detect the more specific association. However, most of the indicators are relatively general (hospitalizations, any anomaly), and more specific indicators (systolic blood pressure) could have any number of consequences for adult health. And, since in this report, we are interested in the very general outcomes of self-rated health and allostatic load, it seems appropriate to use the general indicator of health problems. Second, adding the indicators together implicitly assumes that each of the indicators is equal in magnitude in relation to an outcome of interest. To gain some purchase on how large this problem might be we compare the fit of two models with respect to self-rated health. In a first, we enter the index that sums the indicators and assumes that their magnitudes are equal in weight. In a second, we enter each of the indicators separately thereby allowing their magnitudes to be estimated separately. If the second model were to fit significantly better than the first, we would conclude that the assumption of equal weighting of the indicators is not justified. When we implement this strategy for SRH in our sample, the chi-square associated with the first model is 396.20, and for the second 388.12. The difference (8.08) evaluated at 8 degrees of freedom (9 for nine separate indicators minus 1 for the composite) is not significant. Thus we find no evidence that equal weighting has led us astray in this particular instance. Another issue is that birthweight is not included in the list of 9 indicators and birthweight has received extensive scrutiny as an early life factor influencing later health. However, one of the indicators was gestational age which is a strong correlate of birthweight. Many studies do not have gestational age but because ours was a prospective study with interviewing during pregnancy it was possible to collect information on the number of days between the last menstrual period and birth. We checked and found gestational age to be a stronger predictor of adult self-rated health than birthweight and therefore used it in our composite measure of child health. Because birthweight is associated with gestational age and because some of the child health indicators we include in our composite are plausibly on the pathway from birthweight to health, birthweight is not independently related to adult health when the composite child health measure is controlled. Given this evidence, we believe we have largely accounted for the birthweight to health association.

**#6) The Use of PARAMED to Assess the Proportion of Race and SES Associations that Traits Explain in Allostatic Load.**

Our main tests associated with trait variables concerned 1) whether such traits showed significant direct effects on self-rated health and allostatic load and 2) whether they render coefficients representing race and adult socioeconomic status non-significant. Results in Tables 2 and 3 of the text show that neither of these requirements of a trait explanation were met. As an additional descriptive fact, we also wished to quantify how much of the race and adult SES associations were explained by any of the trait variables. In the linear regression analysis of self-rated health, it was possible to do this by comparing how much the race and adult SES coefficients dropped with the addition of trait variables. However when using non-linear models, it is problematic to calculate how much of an association is explained by reporting the proportionate drop in a coefficient from before to after a potential explanatory variable is added to an equation. As a result, we employ the Stata-supported program PARAMED (Emsley and Liu, 2013) which allows an appropriate assessment of the percent of an association that is accounted for when the dependent variable is a count variable and analyzed via negative binomial regression. To our knowledge, no software is designed to address a non-linear model that is supported within multiple imputation (including PARAMED). Given this, we extracted each of the 25 multiply imputed data sets and ran the relevant PARAMED program on each. We then averaged the percent of the race and adult SES associations accounted for by traits across the 25 data sets. Based on this approach we reported that cognitive ability was the trait that explained the largest proportion of the race/allostatic load association (12.4%) and that trustworthy/self-controlled was the trait that explained the largest proportion of the adult SES/allostatic association (10.9%). We also estimated these proportions in a complete case analysis and found the proportions explained by traits to be quite small (race 11.0% and adult SES 5.5%).

#7) **Potential Indirect Effects of Traits through Adult SES**

The main question we address in this paper is whether traits can explain health inequalities by race and SES.  Another question that could be addressed is whether traits plausibly indirectly affect health through adult SES.  Such a pattern of indirect effects could be observed even if traits fail to account for health inequalities.  Because of this, we tested for the significance of indirect effects of traits on SRH and AL through adult SES.  We used the Stata-supported programs of KHB (Kohler, Carlson, and Holm 2011) for SRH and PARAMED (as discussed above) for AL.  Since neither of these programs is supported in the multiple imputation program of Stata it was necessary to run analyses separately on each of the 25 imputed data sets and then combine the results from them.  As reported in the text there were significant indirect effects of all five trait variables on SRH through adult SES.  Concerning AL the only significant indirect effect was for cognitive ability.  As reported in the text the magnitude of the indirect effects are modest both in terms of effect sizes and in terms of comparisons to the effect of adult SES.   The largest indirect effect -- a 50% difference in a person’s ranking on cognitive ability -- is associated with a .2 difference in SRH or an effect size of only .21.  The effect of adult SES (controlling for traits) ranges from 3.6 (cognitive ability) to 9.7 (mastery) times greater than for the indirect effects associated with the traits. We indicate in the text that these indirect effects are not the focus of our paper but could be usefully pursued in future research. We point out that an indirect-effects-only pattern of results is incompatible with the claim that traits explain health inequalities.

**#8) Tables and Figures Showing Results that Support Statements in the Text and that Test Robustness through Sensitivity Analyses**

**Tables S5 – S7: Race, Gender, and SES Interactions.** In the main text, we model race, gender, and SES as main effects. Here we show results examining interactions. Tables S5 and S6 provide evidence concerning race and SES health inequalities for females and males considered separately for both self-rated health and allostatic load. Results show significant racial inequalities (Table S5) and SES inequalities (Table S6) in SRH and AL for both men and women. Table S7 focuses on whether there are health inequalities by SES within White and Black groups. The relationship between SES and AL within the self-identified Black group does not reach statistical significance although lower SES groups do have higher counts of AL than high SES groups. In all other instances, significant SES inequalities are evident in White and Black groups for SRH and the White group for AL. More importantly, in no instance did we find significant interaction between race and sex (Table S5), SES and sex (Table S6), or race and SES (Table S7) on either SRH or AL. Given these results, we model sex, race, and SES as main effects in the tables presented in the main manuscript.

Tables S5 – S7 About Here

**Table S8: Age and Sex-Adjusted Associations Between Cognitive and Non-Cognitive Traits and Health Outcomes.** In the main text, we show age and sex-adjusted associations between cognitive and non-cognitive traits and the outcomes of self-rated health and allostatic load by quartiles of the traits that are significantly associated with the outcomes. Table S8 shows the results for all the traits as continuous variables. As described in the main text 5 of 10 associations are significant – cognitive ability, trustworthy dependable, and mastery for self-rated health and cognitive ability and trustworthy/self-controlled for allostatic load.

Table S8 About Here

**Table S9: Association Between Childhood Assessed Cognitive and Non-Cognitive Traits and Adult Socioeconomic Status.** In the main text, we describe and show (Figure 3) associations between quartiles of cognitive and non-cognitive traits and self-rated health and allostatic load. Here we show results with all cognitive and non-cognitive traits in continuous form along with the effects of age, sex, race/ethnicity, and childhood SES. As reported in the text each trait predicts adult attainment above and beyond controls. When all five traits are entered together cognitive ability, trustworthy/self-controlled, and self-esteem are significantly, independently associated with higher levels of adult SES.

Table S9 About Here

**Table S10:** **Regression Analyses Showing Associations Between Race and Cognitive and Non-Cognitive Traits.** Results in Table S9 support statements made in the text concerning the association between race and traits. As the Table shows and the text reports two of the five traits (cognitive ability and trustworthy/self-controlled) show associations consistent with the possibility that traits explain racial disparities in SRH and AL whereas three others (valued cognitive traits, mastery, and self-esteem) do not as they are related in a direction opposite to what would need to be true to explain such inequities (2 non-significantly and 1 significantly – self-esteem). Because two are in the expected direction it is important to determine whether and to what extent they account for racial inequities in self-rated health and allostatic load.

Table S10 About Here

**Table S11: The Importance of SES Measurement in Understanding the Role of Traits in Explaining Health Inequalities.** As indicated in the text the only other study we know of that has prospectively collected cognitive and non-cognitive traits in childhood along with health in midlife is one using the 1958 National Child Development Study from the United Kingdom (Carter et al. 2019). That study did not have racial variation or measures based on biomarkers thereby precluding a comparison of our results to theirs concerning those variables. The study did include three binary indicators of SES, father’s manual versus non-manual occupation when the participant was 16, education assessed by a distinction between those who pursued education beyond minimum leaving age and those who did not, and adult occupation also assessed via a distinction between manual and non-manual. Measured in this way the study reported effects of cognitive and non-cognitive traits on self-rated health that were independent of SES. Additionally, the effects of SES especially for the binary indicator of education were reduced when traits were entered. Referring to Table 1 in the text that specifies the patterns that would be consistent with different conclusions about trait explanations for health inequalities these findings align with row 1 which indicates support for such explanations. Accordingly, the Carter et al. (2019), paper concludes that non-cognitive traits are a "potentially valuable target for policies aiming to ameliorate the production of health inequalities." However, we have known for some time that associations between SES and health are characterized by step-by-step gradients thereby ensuring that valuable information is lost by dichotomizing measures as occurred with the three indicators used in this study. Additionally, the study captures childhood SES at only a single point in time and with only a single indicator (parental occupation) and excludes assessments of income and wealth entirely.

To evaluate whether the measurement of SES might matter to conclusions drawn, we mimicked the measurement in the Carter et al. paper by dichotomizing parental occupation at age 16, educational attainment (high school or less versus some college or more) as well as adult occupational standing. Further to be consistent with Carter et al. we also excluded income and wealth. Table S10 shows that had we measured SES in the same truncated way that Carter et al., did our data would have shown precisely the same pattern that theirs did. Equation 1 of the Table shows the effect of SES indicators on self-rated health before controlling traits. Equations 2-6 add cognitive ability and each of the 5 non-cognitive measures separately, equation 7 shows the results of all traits entered together and equation 8 further adds additional covariates. As the Table shows each trait is directly and independently associated with self-rated health when SES as Carter et al. (2019) operationalized it, is controlled. When all traits are considered together with controls in equation 8, cognitive ability and trustworthy/self-controlled remain significant independent predictors. Like Carter et al. our results show that dichotomous education becomes non-significant as traits are controlled but that dichotomized adult occupation remains significant (equations 7 and 8). This correspondence in results when the measurement of SES is similar, strongly suggests that the operationalization of SES matters for the different conclusions that Carter et al. arrive at. The best answer to the question of the role of traits in explaining health inequalities will come from studies that more comprehensively measure SES in child and adult life.

Table S11 About Here

**Tables S12 and S13 Showing SES Components Entered Separately and in Absolute Rather than Relative Form.** As described above, we chose a composite measure of SES and implemented a relative (ranking compared to others) rather than an absolute (keeping variable in the original metric of dollars years of education) approach. Tables 12 and S13 show results using separate measures of SES dimensions that are entered in their original absolute metric. As Table S12 shows education income and wealth are independently associated with self-rated health when controlled for one another. Occupational standing is not but is if it is entered alone. Table S13 shows that wealth is the SES dimension that is uniquely associated with allostatic load when other dimensions are controlled. However, our main questions are not about these unique effects only but also on what the SES dimensions share. In this regard, the main question we seek to answer is whether traits can account for health inequalities by race and SES. As such, the key issues in these sensitivity analyses are whether 1) any traits are independently associated with health when the four dimensions of adult SES are separately controlled and 2) whether the four separate SES variables entered as a block are significantly associated with health when added to an equation that controls all of the traits. As the Tables show none of the traits are independently associated with either self-rated health or allostatic load when the four SES variables are controlled. We also checked whether the four SES variables were significantly associated with the health outcomes by implementing a four-degree-of-freedom joint test of their significance. This is important because the tests shown in the table are for the unique effects of each dimension of SES (controlling the other dimensions) but we are also interested in the joint effects. The four degree of freedom joint tests shows that for both self-rated health (p<.001) and allostatic load (P<.05) the four SES dimensions taken together have a significant association with the health outcomes with traits and other variables controlled. These results directly mimic the conclusions we draw in the analyses presented in the main paper where we use the composite measure of SES.

Table S12 and S13 About Here

**Table S14 and Figure S2 Analyses Showing Significant Interactions Between Race and Cognitive Ability, Race and Trustworthy/self-controlled, and Race and Mastery on Self-Rated Health**. As reported in the text 3 of 20 tested interactions (2 independent variables – adult SES and race by 5 traits by 2 outcomes) were statistically significant at p<.05. Given the number of tests we are not inclined to strongly interpret the interactions that did emerge. However, we did want to report them as there is evidence to suggest that Black people receive fewer health returns to education and income (Assari 2018, Bartle-Haring and Whiting 2022). Our results suggest reduced returns to what many regard as beneficial traits and thereby cohere to some extent with the literature on returns to education and income. Most important for the main concern of the current paper is whether they suggest a role for traits that would be consistent with the trait explanations. In this regard, as explained in the text, we could have observed that at high levels of beneficial traits SES and racial gaps in health would not be observed, that beneficial levels of such traits would allow the erasure of such health inequalities. However, what we in fact found in the few instances in which interactions were significant was just the opposite, racial inequities were larger when beneficial traits were higher. Thus, even if we did choose to interpret the 3 of 20 interactions we found to be significant the evidence they would bring would not be supportive of the trait explanations we test.

Table S14 and Figure S2 About Here

**Tables S13 and S14:** **Complete Case Results Showing Multiple and Negative Binomial Regression Equations Assessing Whether Cognitive and/or Non-Cognitive Traits Account for Race and SES Inequalities in Self-Rated Health and Allostatic Load.** Our approach to missing data involved the use of multiple imputation. Tables S13 and S14 present complete case analyses that involve no imputation at all. Each column in the tables uses all valid cases for the variables included in that equation and as a consequence, the number of cases for each equation differs. Our goal in these sensitivity analyses was to determine whether our approach to missing data would change conclusions, that is would we see a sharp diminution in the coefficients for adult SES and race as cognitive and non-cognitive traits were entered and would we see independent effects of the traits on health. The complete case analyses are very closely aligned with the multiple imputation results, sharp declines in the adult SES and race coefficients do not occur and only one of the trait variables (trustworthy dependable) showed an independent effect on health in 10 tests (5 traits * 2 health outcomes). Thus the results of the complete case analysis closely mirror the results of the multiple imputation analyses.

Table S15 and S16 About Here

References

Allison, Paul D. 2002. *Missing Data.* Thousand Oaks, CA: Sage.

Assari, Shervin. 2018. “Health Disparities Due to Diminished Return Among Black Americans: Public Policy Solutions.” *Social Issues and Policy Review* 12(1):112–145.

Bartle-Haring, Suzanne, and Riley Whiting. 2022. “Structural Racism and the Education Gradient for Early All-Cause Mortality.” *SSM-Population Health* 17:101076.

Bollen, Kenneth A., and Shawn Bauldry. 2011. “Three Cs in Measurement Models: Causal Indicators, Composite Indicators, and Covariates.” *Psychological Methods* 16(3):265–284.

Brady, David, Michaela Curran, and Richard M. Carpiano. 2023. “A Test of the Predictive Validity of Relative Versus Absolute Income for Self-Reported Health and Well-Being in the United States.” *Demographic Research* 48:775–808.

Carter, Jennifer L., Marcus Richards, Matthew Hotopf, and Stephani L. Hatch. 2019. “The Roles of Non-Cognitive and Cognitive Skills in the Life Course Development of Adult Health Inequalities.” *Social Science & Medicine* 232:190–198.

Cirillo, Pamela M., and Barbara A. Cohn. 2015. “Pregnancy Complications and Cardiovascular Disease Death: 50-Year Follow-Up of the Child Health and Development Studies Pregnancy Cohort.” *Circulation* 132(13):1234–1242.

Cohn, Barbara A., Pamela Cirillo, Robert Christianson, B. van den Berg, and P. Siiteri. 2001. “Placental Characteristics and Reduced Risk of Maternal Breast Cancer.” *Journal of the National Cancer Institute* 93(15):1133–1140.

Cohn, Barbara A., Mary S. Wolff, Pamela M. Cirillo, and R. I. Sholtz. 2007. “DDT and Breast Cancer in Young Women: New Data on the Significance of Age at Exposure.” *Environmental Health Perspectives* 115(10):1406–1414.

Cohn, Barbara A., Pamela M. Cirillo, and Robert E. Christianson. 2010. “Prenatal DDT Exposure and Testicular Cancer: A Nested Case-Control Study.” *Archives of Environmental & Occupational Health* 65(3):127–134.

Emsley, Richard, and Hanhua Liu. 2013. “PARAMED: Stata Module to Perform Causal Mediation Analysis Using Parametric Regression Models.” *Statistical Software Components* S457581, Boston College Department of Economics.

Heckman, James, Rodrigo Pinto, and Peter Savelyev. 2013. “Understanding the Mechanisms Through Which an Influential Early Childhood Program Boosted Adult Outcomes.” *American Economic Review* 103(6):2052–2086.

Gottfredson, Linda. 2004. "Intelligence: Is It the Epidemiologists’ Elusive ‘Fundamental Cause’ of Social Class Inequalities in Health?" *Journal of Social and Personality Psychology* 86(1):174-199.

Kaiser Permanente. n.d. “Our History.” Retrieved February 17, 2016 (<http://share.kaiserpermanente.org/article/history-of-kaiser-permanente/>).

Kezios, Katrina L., Shakira F. Suglia, David Matthew Doyle, Ezra Susser, Gary Bradwin, Piera Cirillo, Barbara Cohn, Bruce Link, and Pam Factor-Litvak. 2022. “Comparing Different Operationalizations of Allostatic Load Measured in Mid-Life and Their Patterning by Race and Cumulative Life Course Socioeconomic Status.” *Psychoneuroendocrinology* 139:105689.

Kohler, Ulrich, Kristian Bernt Karlson, and Anders Holm. 2011. “Comparing Coefficients of Nested Nonlinear Probability Models.” *Stata Journal* 11(3):420–39.

Krieger, Nancy. 1992. “Overcoming the Absence of Socioeconomic Data in Medical Records: Validation and Application of Census-Based Methodology.” *American Journal of Public Health* 82(5):703–710.

Luo, Lijun, and Ling Wei. 2024. “For Whom Does Education Convey Health Benefits? A Two-Generation and Life Course Approach.” *Journal of Health and Social Behavior* 65(4):596–617.

Mongraw-Chaffin ML, Cirillo PM, Cohn BA. 2010. Preeclampsia and cardiovascular disease death: prospective evidence from the child health and development studies cohort. *Hypertension.* 56(1):166-71.

Moore, Shirley Ann Wilson. 2000. *To Place Our Deeds: The African American Community in Richmond, California, 1910–1963.* Berkeley, CA: University of California Press.

National Health and Nutrition Examination Survey (NHANES). n.d. “National Health and Nutrition Examination Survey.” Retrieved February 17, 2016 (<http://www.cdc.gov/nchs/nhanes.htm>).

Rifai, Nader, Thomas G. Cole, Elizabeth Iannotti, Todd Law, Martin Macke, Robert Miller, David Dowd, and Donald A. Wiebe. 1998. “Assessment of Interlaboratory Performance in External Proficiency Testing Programs with a Direct HDL-Cholesterol Assay.” *Clinical Chemistry* 44:1452–1458.

Rhomberg, Chris. 2004. *No There There: Race, Class, and Political Community in Oakland.* Berkeley, CA: University of California Press.

Self, Robert O. 2003. *American Babylon: Race, Power, and the Struggle for the Postwar City in California.* Princeton, NJ: Princeton University Press.

Smith-Greenaway, Emily, Yingyi Lin, and Abigail Weitzman. 2024. “The Intergenerational Transmission of Health Disadvantage: Can Education Disrupt It?” *Journal of Health and Social Behavior* 65(4):577–595.

Sugiuchi, Hiroshi, Yasuo Uji, Hiroshi Okabe, Takayuki Irie, Koji Uekama, Noboru Kayahara, and Kenichi Miyauchi. 1995. “Direct Measurement of High-Density Lipoprotein Cholesterol in Serum with Polyethylene Glycol-Modified Enzymes and Sulfated Alpha-Cyclodextrin.” *Clinical Chemistry* 41:717–723.

van den Berg, B. J., Robert E. Christianson, and F. W. Oechsli. 1988. “The California Child Health and Development Studies of the School of Public Health, University of California at Berkeley.” *Pediatric and Perinatal Epidemiology* 2(3):265–282.

Wang, Erica T., Pamela M. Cirillo, Eric Vittinghoff, Kirsten Bibbins-Domingo, Barbara A. Cohn, and Marcelle I. Cedars. 2011. “Menstrual Irregularity and Cardiovascular Mortality.” *Journal of Clinical Endocrinology & Metabolism* 96(1):E114–E118.

| Table S1. Time-points of prospective observation for the CHDS Disparities Study Sample by Race and Gender. | | | | | | | | | | | | | | | |
| --- | --- | --- | --- | --- | --- | --- | --- | --- | --- | --- | --- | --- | --- | --- | --- |
|  | **All** | | | **non-Black and Male** | | | **Black and Male** | | | **non-Black and Female** | | | **Black and Female** | | |
|  |  | Total | |  | Total | |  | Total | |  | Total | |  | Total | |
| Time-points of observation | n | N | % | n | N | % | n | N | % | n | N | % | n | N | % |
| Four time points |  |  |  |  |  |  |  |  |  |  |  |  |  |  |  |
| Birth, Ages 5, 9-11, and 15-17 | 420 |  |  | 150 |  |  | 47 |  |  | 156 |  |  | 67 |  |  |
| Total |  | 420 | 69.4 |  | 150 | 84.7 |  | 47 | 37.3 |  | 156 | 88.6 |  | 67 | 53.2 |
| Three time points |  |  |  |  |  |  |  |  |  |  |  |  |  |  |  |
| Birth, Ages 9-11 and 15-17 | 54 |  |  | 25 |  |  | 3 |  |  | 20 |  |  | 6 |  |  |
| Birth, Ages 5 and 9-11 | 42 |  |  | 0 |  |  | 17 |  |  | 0 |  |  | 25 |  |  |
| Birth, Ages 5 and 15-17 | 2 |  |  | 2 |  |  | 0 |  |  | 0 |  |  | 0 |  |  |
| Total |  | 98 | 16.2 |  | 27 | 15.3 |  | 20 | 15.9 |  | 20 | 11.4 |  | 31 | 24.6 |
| Two time points |  |  |  |  |  |  |  |  |  |  |  |  |  |  |  |
| Birth, Age 5 | 49 |  |  | 0 |  |  | 42 |  |  | 0 |  |  | 7 |  |  |
| Birth, Ages 9-11 | 37 |  |  | 0 |  |  | 17 |  |  | 0 |  |  | 20 |  |  |
| Birth, Ages 15-17 | 1 |  |  | 0 |  |  | 0 |  |  | 0 |  |  | 1 |  |  |
| Total |  | 87 | 14.4 |  | 0 | 0.0 |  | 59 | 46.8 |  | 0 | 0.0 |  | 28 | 22.2 |
| Total sample |  | 605 | 100.0 |  | 177 | 100.0 |  | 126 | 100.0 |  | 176 | 100.0 |  | 126 | 100.0 |

**Table S2.** Selected baseline characteristics comparing contacted subjects who participated compared to those who did not by race/ethnicity^1^.

|  | Black, contacted,  did not participate  (N=699) | | Black, contacted,  participated  (N=252) | |  | White, contacted,  did not participate  (N=282) | | White, contacted,  participated  (N=317) | |  |
| --- | --- | --- | --- | --- | --- | --- | --- | --- | --- | --- |
| **Characteristics at baseline**^2^ | N | % | N | % | *p* | N | % | N | % | *p* |
| **Mother's age** |  |  |  |  | 0.62 |  |  |  |  | 0.11 |
| <20 years | 42 | 6.0 | 11 | 4.4 |  | 15 | 5.3 | 7 | 2.2 |  |
| 20-29 years | 390 | 56.0 | 143 | 57.0 |  | 157 | 55.7 | 174 | 55.3 |  |
| 30+ years | 265 | 38.0 | 97 | 38.6 |  | 110 | 39.0 | 134 | 42.5 |  |
| Missing | 2 | ----- | 1 | ----- |  | 0 | ----- | 2 | ----- |  |
| **Mother's education** |  |  |  |  | 0.27 |  |  |  |  | 0.20 |
| Less than high school | 181 | 26.5 | 57 | 22.9 |  | 30 | 10.6 | 23 | 7.3 |  |
| High school/trade school | 272 | 39.9 | 95 | 38.1 |  | 123 | 43.6 | 130 | 41.0 |  |
| College | 229 | 33.6 | 97 | 39.0 |  | 129 | 45.8 | 164 | 51.7 |  |
| Missing | 17 | ----- | 3 | ----- |  | 0 | ----- |  | ----- |  |
| **Mother's employment** |  |  |  |  | 0.65 |  |  |  |  | 0.46 |
| Homemaker | 380 | 57.1 | 149 | 60.3 |  | 176 | 63.5 | 190 | 60.9 |  |
| Professional/managerial | 48 | 7.2 | 12 | 4.9 |  | 33 | 11.9 | 37 | 11.9 |  |
| Secretarial, clerical | 143 | 21.5 | 48 | 19.4 |  | 55 | 19.9 | 73 | 23.4 |  |
| Service workers, laborers | 89 | 13.3 | 35 | 14.2 |  | 13 | 4.7 | 10 | 3.2 |  |
| Other | 6 | 0.9 | 3 | 1.2 |  | 0 | 0.0 | 2 | 0.6 |  |
| Missing | 33 | ----- | 5 | ----- |  | 5 | ----- | 5 | ----- |  |
| **Family income (quartiles)** |  |  |  |  | 0.84 |  |  |  |  | 0.34 |
| <$5,000 | 192 | 32.6 | 72 | 33.3 |  | 54 | 21.1 | 54 | 18.8 |  |
| $5,000-$6,999 | 193 | 32.8 | 73 | 33.8 |  | 69 | 27.0 | 93 | 32.4 |  |
| $7,000-$8,999 | 103 | 17.5 | 32 | 14.8 |  | 73 | 28.5 | 67 | 23.3 |  |
| ≥$9,000 | 101 | 17.1 | 39 | 18.1 |  | 60 | 23.4 | 73 | 25.5 |  |
| Missing | 110 | ----- | 36 | ----- |  | 26 | ----- | 30 | ----- |  |
| **Offspring birth weight** |  |  |  |  | 0.39 |  |  |  |  | 0.53 |
| ≤2,940 g | 174 | 24.9 | 70 | 27.8 |  | 45 | 16.0 | 61 | 19.2 |  |
| 2,941-3276 g | 197 | 28.2 | 69 | 27.4 |  | 55 | 19.5 | 63 | 19.9 |  |
| 3277-3584 g | 153 | 21.9 | 62 | 24.6 |  | 72 | 25.5 | 86 | 27.1 |  |
| ≥3585 g | 175 | 25.0 | 51 | 20.2 |  | 110 | 39.0 | 107 | 33.8 |  |
| **Father's age** |  |  |  |  | 0.79 |  |  |  |  | 0.43 |
| <27 years | 188 | 27.7 | 63.0 | 25.7 |  | 76 | 27.2 | 72 | 22.9 |  |
| 27-32years | 196 | 28.9 | 70 | 28.6 |  | 95 | 34.1 | 109 | 34.6 |  |
| 33+ years | 295 | 43.4 | 112 | 45.7 |  | 108 | 38.7 | 134 | 42.5 |  |
| Missing | 20 | ----- | 7 | ----- |  | 3 | ----- | 2 | ----- |  |
| **Father's education** |  |  |  |  | 0.27 |  |  |  |  | 0.32 |
| Less than high school | 210 | 32.2 | 68 | 28.3 |  | 32 | 11.5 | 30 | 9.5 |  |
| High school/trade school | 257 | 39.4 | 91 | 37.9 |  | 86 | 30.9 | 85 | 26.9 |  |
| College | 185 | 28.4 | 81 | 33.8 |  | 160 | 57.6 | 201 | 63.6 |  |
| Missing | 47 | ----- | 12 | ----- |  | 6 | ----- | 1 | ----- |  |
| **Father's employment** |  |  |  |  | 0.11 |  |  |  |  | 0.11 |
| Professional/managerial | 53 | 8.0 | 16 | 6.6 |  | 122 | 43.4 | 167 | 53.4 |  |
| Clerical, sales | 111 | 16.7 | 52 | 21.3 |  | 41 | 14.6 | 46 | 14.7 |  |
| Craftsmen, operative, foremen | 256 | 38.6 | 82 | 33.6 |  | 87 | 31.0 | 71 | 22.7 |  |
| Service workers, laborers | 235 | 35 | 90 | 37 |  | 28 | 10 | 27 | 8.6 |  |
| Other | 3 | 0.5 | 4 | 1.6 |  | 3 | 1.0 | 2 | 0.6 |  |
| Unemployed | 5 | 0.8 | 0 | 0.0 |  | 0 | ----- | 0 | ----- |  |
| Missing | 36 | ----- | 8 | ----- |  | 1 | ----- | 4 | ----- |  |

^1^ 83 cohort participants whose mothers were categorized as ‘other’ race/ethnicity (22 Latino, 60 Asian, 1 mixed-race are not included in the Table. Of these, 36 participants agreed to participate. No significant differences in baseline characteristics (p<0.05) were observed between those who agreed to participate and those who did not.

^2^baseline parental and participant characteristics are from the time point of the participant’s birth.

**Table S3.** Comparing selected variables from NHANES 2011-2012 with CHDS-Disparities Study, stratified by race (restricted to Non-Hispanic White and Non-Hispanic Black).

|  | Total Sample^1^ | |  | NHANES (2011-2012)^1^ | | |  | CHDS-Disparities Study^1^ | | |  |  |
| --- | --- | --- | --- | --- | --- | --- | --- | --- | --- | --- | --- | --- |
|  | **NHANES** | **CHDS-Disparities Study^1^** |  | **White** | **Black** |  |  | **White** | **Black** |  |  |  |
| Variables | **mean ± sd** | **mean ± sd** | **p_data_^2^** | **mean ± sd** | **mean ± sd** | **p_race_^3^** |  | **mean ± sd** | **mean ± sd** | **p_race_^3^** | **p_totrace_^4^** | **p_disparity_^5^** |
| BMI | 30.8 ± 7.46 | 30.0 ± 6.90 | 0.07 | 29.9 ± 6.49 | 32.0 ± 8.38 | <0.01 |  | 28.4 ± 5.97 | 31.9 ± 7.45 | <0.01 | <0.01 | 0.14 |
| *BMI males* | 29.5 ± 6.31 | 29.4 ± 5.65 | 0.95 | 29.2 ± 5.98 | 29.8 ± 6.73 | 0.52 |  | 28.5 ± 4.47 | 30.5 ± 6.65 | <0.01 | 0.02 | 0.21 |
| *BMI females* | 32.1 ± 8.22 | 30.5 ± 7.92 | 0.03 | 30.5 ± 6.92 | 34.0 ± 9.23 | <0.01 |  | 28.2 ± 6.65 | 33.3 ± 7.98 | <0.01 | <0.01 | 0.28 |
| Hemoglobin A1c | 5.82 ± 1.21 | 5.87 ± 1.14 | 0.54 | 5.67 ± 0.99 | 6.00 ± 1.43 | <0.01 |  | 5.64 ± 0.81 | 6.24 ± 1.50 | <0.01 | <0.01 | 0.11 |
| Blood pressure |  |  |  |  |  |  |  |  |  |  |  |  |
| *Systolic* | 123 ± 18.2 | 125 ± 16.4 | 0.18 | 120 ± 14.3 | 128 ± 21.3 | <0.01 |  | 122 ± 13.8 | 129 ± 18.1 | <0.01 | <0.01 | 0.97 |
| *Diastolic* | 75.9 ± 11.1 | 74.6 ± 10.6 | 0.08 | 75.0 ± 10.7 | 77.0 ± 11.6 | 0.07 |  | 72.8 ± 10.1 | 76.8 ± 10.8 | <0.01 | <0.01 | 0.20 |
| Self-Rated Health | **N (%)** | **N (%)** |  | **N (%)** | **N (%)** |  |  | **N (%)** | **N (%)** |  |  |  |
| *Excellent, very good, good* | 290 (78%) | 452 (82%) | <0.01 | 168 (81%) | 122 (74%) | 0.03 |  | 269 (88%) | 183 (75%) | <0.01 | <0.01 | 0.16 |
| *Fair or poor* | 82 (22%) | 98 (18%) |  | 40 (19%) | 42 (26%) |  |  | 38 (12%) | 60 (25%) |  |  |  |

^1^Total sample for NHANES (N=372) and CHDS-Disparities Study (N=552) restricted to those who self-report race as White or Non-Hispanic black; total number of White NHANES participants = 227; total number of Black NHANES participants = 188; total number of White DISPAR participants = 308 (self-reported race); total number of Black DISPAR participants = 244 (self-reported race).

^2^p-values were obtained from univariate two-sample T-tests comparing means of variables across datasets for continuous variables and chi-square tests for dichotomous variables

^3^p-values were obtained from univariate two-sample T-tests comparing means of variables across race groups within each dataset and chi-square tests for dichotomous variables

^4^p-values were obtained from regression models that included terms for dataset, race, and a dataset*race cross-product term. p_totrace_ is the p-value of the race term and tests for an overall difference in variables within race groups (type I sum of squares p-value reported for linear regression analyses and Wald tests of individual effects p-values are reported for logistic regression analyses).

^5^p-values were obtained from regression models that included terms for dataset, race, and a dataset*race cross-product term. p_disparity_ is p-value of the cross-product term and tests for a difference in variables within race between datasets (ie. looking to see if apparent disparities are the same between the two datasets). Type I sum of squares p-value reported for linear regression analyses and Wald tests of individual effects p-values are reported for logistic regression analyses.

**Table S4.** Comparing selected variables from NHANES 2011-2012 with CHDS-Disparities Study, stratified by level of educational attainment (high school (HS) graduate or less, some college, and college graduate) and restricted to only non-Hispanic White and non-Hispanic black participants.

|  | Total Sample^1^ | |  | NHANES (2011-2012)^1^ | | | |  | CHDS-Disparities Study | | | | |  |  |
| --- | --- | --- | --- | --- | --- | --- | --- | --- | --- | --- | --- | --- | --- | --- | --- |
|  | **NHANES** | **CHDS-Disparities Study^1^** |  | **≤ HS** | **Some college** | **College grad** |  |  | **≤ HS** | **Some college** | | **College grad** |  |  |  |
| Variables | **mean ± sd** | **mean ± sd** | **p_dataset_^2^** | **mean ± sd** | **mean ± sd** | **mean ± sd** | **p_educ_^3^** |  | **mean ± sd** | | **mean ± sd** | **mean ± sd** | **p_educ_^3^** | **p_toteduc_^4^** | **p_disparity_^5^** |
| BMI | 30.8 ± 7.46 | 30.0 ± 6.90 | 0.07 | 30.4 ± 6.93 | 32.2 ± 8.11 | 29.9 ± 7.39 | 0.04 |  | 28.9 ± 6.36 | | 31.6 ± 8.23 | 29.8 ± 5.88 | <0.01 | <0.01 | 0.95 |
| *BMI males* | 29.5 ± 6.31 | 29.4 ± 5.65 | 0.95 | 28.0 ± 4.94 | 31.5 ± 7.39 | 30.2 ± 6.89 | <0.01 |  | 29.3 ± 5.32 | | 31.8 ± 6.97 | 27.8 ± 3.98 | <0.01 | <0.01 | 0.02 |
| *BMI females* | 32.1 ± 8.22 | 30.5 ± 7.92 | 0.03 | 33.5 ± 7.83 | 32.9 ± 8.65 | 29.6 ± 7.79 | 0.01 |  | 30.5 ± 6.49 | | 31.4 ± 9.43 | 29.9 ± 7.70 | 0.48 | 0.03 | 0.22 |
| Hemoglobin A1c | 5.82 ± 1.21 | 5.87 ± 1.14 | 0.54 | 5.84 ± 1.17 | 5.91 ± 1.00 | 5.69 ± 1.48 | 0.39 |  | 5.98 ± 1.46 | | 6.12 ± 1.29 | 5.65 ± 0.71 | <0.01 | <0.01 | 0.47 |
| Blood pressure |  |  |  |  |  |  |  |  |  | |  |  |  |  |  |
| *Systolic* | 123 ± 18.2 | 125 ± 16.4 | 0.18 | 126 ± 19.8 | 124 ± 18.0 | 118 ± 14.2 | <0.01 |  | 126 ± 15.9 | | 126 ± 17.5 | 123 ± 15.8 | 0.09 | <0.01 | 0.20 |
| *Diastolic* | 75.9 ± 11.1 | 74.6 ± 10.6 | 0.08 | 76.3 ± 11.1 | 76.0 ± 11.6 | 75.1 ± 10.6 | 0.69 |  | 75.1 ± 10.4 | | 75.0 ± 10.9 | 73.9 ± 10.5 | 0.50 | 0.35 | 0.99 |
| Self-Rated Health | **N (%)** | **N (%)** |  | **N (%)** | **N (%)** | **N (%)** |  |  | **N (%)** | | **N (%)** | **N (%)** |  |  |  |
| *Excellent,*  *very good, good* | 290 (78%) | 452 (82%) | <0.01 | 113 (71%) | 84 (76%) | 93 (92%) | <0.01 |  | 114 (74%) | | 124 (77%) | 212 (92%) | <0.01 | <0.01 | 0.98 |
| *Fair or poor* | 82 (22%) | 98 (18%) |  | 46 (29%) | 27 (24%) | 9 (9%) |  |  | 41 (26%) | | 38 (23%) | 19 (8%) |  |  |  |

^1^Total sample when restricted to participants who self-reported race/ethnicity as Black or White: NHANES (N=372) and CHDS-Disparities Study (N=552); in this restricted analysis, total number of NHANES participants by levels of educational attainment is as follows: ≤HS = 180 participants, some college = 124 participants, college graduate = 111 participants; total number of DISPAR participants by levels of educational attainment: ≤HS = 156 participants, some college = 162 participants, college graduate = 232 participants.

^2^p-values were obtained from univariate two-sample T-tests comparing means of variables across datasets for continuous variables and chi-square tests for dichotomous variables

^3^p-values were obtained from ANOVA tests comparing means of variables across education groups within each dataset and chi-square tests for dichotomous variables

^4^p-values were obtained from regression models that included terms for dataset, education, and a dataset* education cross-product term. p_toteduc_ is the p-value of the education term and tests for an overall difference in variables within education groups (type I sum of squares p-value reported for linear regression analyses and Wald tests of individual effects p-values are reported for logistic regression analyses).

^5^p-values were obtained from regression models that included terms for dataset, education, and a dataset* education cross-product term. p_disparity_ is p-value of the cross-product term and tests for a difference in variables within education between datasets (ie. looking to see if apparent disparities are the same between the two datasets). Type I sum of squares p-value reported for linear regression analyses and Wald tests of individual effects p-values are reported for logistic regression analyses.

| Table S6: Mean Values of Self-Rated Health (SRH) and Counts of Allostatic Load (AL) within Quartiles of Adult SES among Women and Men | | | | | | | | | |
| --- | --- | --- | --- | --- | --- | --- | --- | --- | --- |
|  | Self-Rated Health  (1= Poor - 5= Excellent) | | | |  | Allostatic Load  (Count of Allostatic Load Indicators) | | | |
|  | Lowest Quartile Adult SES | 2^nd^ Quartile Adult SES | 3rd Quartile Adult SES | Highest Quartile Adult SES |  | Lowest Quartile Adult SES | 2^nd^ Quartile Adult SES | 3rd Quartile Adult SES | Highest Quartile Adult SES |
|  |  |  |  |  |  |  |  |  |  |
| Female | 3.06  (N=70) | 3.27  (N=77) | 3.43  (N=89) | 4.01^a^  (N=64) |  | 2.89  (N=47) | 2.41  (N=44) | 1.98  (N=54) | 1.49^d^  (N=45) |
| Male | 2.97  (N=79) | 3.39  (N=74) | 3.75  (N=61) | 3.91^b,c^  (N=79) |  | 3.48  (N=48) | 2.82  (N=52) | 2.39  (N=46) | 2.25^e,f^  (N=64) |
| ^a^n Adult SES within female for SRH, P<.001; ^b^ Adult SES within male for SRH, P< .001,’ ^c^ Interaction of adult SES and sex on SRH, n.s.; Adult SES within female for AL, P<.05; Adult SES within male for AL, P< .01; Interaction of adult SES and sex on AL, n.s. | | | | | | | | | |

| Table S5: Mean Values of Self-Rated Health (SRH) and Counts of Allostatic Load (AL) in Black Women, Black Men, White Women, and White Men | | | | | |
| --- | --- | --- | --- | --- | --- |
|  | | | | | |
|  | Self-Rated Health  (1= Poor - 5= Excellent) | |  | Allostatic Load  (Count of Allostatic Load Indicators) | |
|  | Self-identified as Black | Self-identified as White |  | Self-identified as Black | Self-identified as White |
|  |  |  |  |  |  |
| Female | 3.10  (N=121) | 3.72^a^  (N=154) |  | 3.03  (N=62) | 1.81^d^  (110) |
| Male | 3.27  (N=122) | 3.70^b.c^  (N=155) |  | 3.35  (N=77) | 2.21^e,f^  (N=117) |
| ^a^ Race within female for SRH, P<.001; ^b^ Race within male for SRH, P< .001 , ^c^ Interaction of race and sex on SRH, n.s.; Race within female for AL, P<.001 ; Race within male for AL, P< .001; Interaction of race and sex on AL, n.s.. | | | | | |
|  | | | | | |

| Table S7: Mean Values of Self-Rated Health (SRH) and Counts of Allostatic Load (AL) within Quartiles of Adult SES among Black and White People | | | | | | | | | |
| --- | --- | --- | --- | --- | --- | --- | --- | --- | --- |
|  | Self-Rated Health  (1= Poor - 5= Excellent) | | | |  | Allostatic Load  (Count of Allostatic Load Indicators) | | | |
|  | Lowest Quartile Adult SES | 2^nd^ Quartile Adult SES | 3rd Quartile Adult SES | Highest Quartile Adult SES |  | Lowest Quartile Adult SES | 2^nd^ Quartile Adult SES | 3rd Quartile Adult SES | Highest Quartile Adult SES |
|  |  |  |  |  |  |  |  |  |  |
| Self-Identified  As Black | 3.02  (N=89) | 3.11  (N=70) | 3.11  (N=50) | 3.92^a^  (N=32) |  | 3.28  (N=56) | 3.54  (N=35) | 3.03  (N=30) | 2.61^d^  (N=18) |
| Self-identified as White | 3.04  (N=55) | 3.61  (N=66) | 3.85  (N=88) | 4.02^b,c^  (N=100) |  | 2.77  (N=36) | 2.04  (N=51) | 1.79  (N=62) | 1.83^e,f^  (N=78) |
| ^a^ Adult SES within Black people for SRH, P<.001; ^b^ Adult SES within White people for SRH, P<.001, ^c^ Interaction of adult SES and race on SRH, n.s.; ^d^ Adult SES within Black people for AL, n.s.; ^e^ Adult SES within White people for AL, P< .05; ^f^ Interaction of adult SES and race on AL, n.s. | | | | | | | | | |

| Table S8: Age and Sex Adjusted Regression Analyses Showing Associations Between Cognitive and Non-Cognitive Traits and Self-rated Health (Ordinary Least Squares) and Allostatic Load (Negative Binomial): Regression Coefficients (Standard Errors) | | | | | | | | | | | |
| --- | --- | --- | --- | --- | --- | --- | --- | --- | --- | --- | --- |
|  | Self-Rated Health  (N=603) | | | | |  | Allostatic Load  (N=400) | | | | |
| Female | -.005  (.078) | -.096  (.080) | -.081  (.080) | -.068  (.080) | -.053  (080) |  | -.261** (.089) | -.192* (.090) | -.216* (.091) | -.200* (.090) | -.208* (.091) |
| Age | -.075*  (.033) | -.035  (.032) | -.016  (.032) | -.015  (.032) | -.019  (.032) |  | .018 (.036) | -.013 (.036) | -.023 (.035) | -.029 (.035) | -.028 (.035) |
| Cognitive Ability | 1.422***  (.218) |  |  |  |  |  | -.103*** (.256) |  |  |  |  |
| Trustworthy/Self-Controlled |  | .871***  (.200) |  |  |  |  |  | -.596* (.231) |  |  |  |
| Valued Non-Cognitive Traits |  |  | .392  (.323) |  |  |  |  |  | .595 (.373) |  |  |
| Mastery |  |  |  | .586*  (.261) |  |  |  |  |  | -.153 (.275) |  |
| Self-esteem |  |  |  |  | .397  (.232) |  |  |  |  |  | -.161 (.276) |
| Constant | 6.558***  (1.581) | 4.601***  (1.568) | 3.940*  (1.604) | 3.846*  (1.584) | 4.151**  (1.574) |  | .595 (1.765) | 2.065 (1.744) | 1.609 (1.797) | 2.513 (1.754) | 2.463 (1.744) |
| *p <.05; ** p<.01; ***p < .001 | | | | | | | | | | | |

| Table S9. Regression Analyses Showing Associations Between Cognitive and Non-Cognitive Traits and Adult SES Attainment: Regression Coefficients (Standard Errors) N= 603 | | | | | | |
| --- | --- | --- | --- | --- | --- | --- |
|  | Eq. 1 | Eq. 2 | Eq. 3 | Eq. 4 | Eq. 5 | Eq. 6 |
| Self-identified Black race | -.036* (.018) | -.060** (.018) | -.074*** (.018) | -.070*** (.017) | -.084*** (.018) | -.044* (.019) |
| Other race/ethnicity | .075** (.027) | .061* (.027) | .066* (.027) | .067* (.027) | .068* (.027) | 071** (.026) |
| Female | .012 (.015) | -.004 (.015) | -.003 (.015) | .001 (.015) | .008 (.015) | .010 (.015) |
| Age | -.007 (.006) | -.001 (.006) | .001 (.006) | .001 (.006) | -.001 (.006) | -.009 (.006) |
| Child SES | .206*** (.041) | .268*** (.039) | .288*** (.039) | .279*** (.039) | .266*** (.039) | .198*** (.041) |
| Cognitive Ability | .279*** (.049) |  |  |  |  | .238*** (.050) |
| Trustworthy/Self-controlled |  | .154*** (.037) |  |  |  | .125** (.037) |
| Valued Non-Cognitive Traits |  |  | .143* (.062) |  |  | .075 (.061) |
| Mastery |  |  |  | .104* (.047) |  | -.010 (.055) |
| Self-esteem |  |  |  |  | .161** (.046) | .110* (.055) |
| Constant | .662* (.301) | .363 (.295) | .223 (.303) | .267 (.299) | .329 (.297) | .545 (.305) |
| *p <.05; ** p<.01; ***p < .001 | | | | | | |

| Table S10. Regression Analyses Showing Associations Between Race and Cognitive and Non-Cognitive Traits (N=603) (Standard Errors) | | | | | |
| --- | --- | --- | --- | --- | --- |
|  | Cognitive Ability | Trustworthy/Self-controlled | Valued Non-Cognitive Traits | Mastery | Self-esteem |
| Self-identified Black race | -.122***  (.01**6**) | -.065**  (.024) | .026  (.015) | .001  (.022) | .086***  (.023) |
| Other race/ethnicity | -.032  (.023) | .026  (.039) | .000  (.022) | -.013  (.029) | -.013  (.031) |
| Age | .030***  (.007) | .014  (.009) | -.002  (.007) | -.013  (.029) | .010  (.009) |
| Female | -.041**  (.013) | .031  (.023) | .021  (.013) | -.006  (.016) | -.049**  (.017) |
| Child SES | .272 ***  (.035) | .093  (.057) | -.040  (.032) | .041  (.043) | .099*  (.047) |
| Constant | -1.091*  (.336) | -.038  (.460) | .929  (.356) | .865  (.498) | .166  (.467) |
| *p <.05; ** p<.01; ***p < .001 | | | | | |

| Table S11. Multiple Regression Equations with SES Dimensions Assessed Separately and in Absolute Metrics. Results test whether Cognitive and/or Non-Cognitive Traits Account for Race and SES Health Inequalities in Self-Rated Health: Regression Coefficients (Standard Errors), N=603. | | | | | | |
| --- | --- | --- | --- | --- | --- | --- |
|  | Eq. 1 | Eq. 2 | Eq. 3 | Eq. 4 | Eq. 5 | Eq. 6 |
| Self- Identified Black Race | -.249**  (.095) | -.262**  (.090) | -.292***  (.090) | -.283**  (.089) | -.306***  (.093) | -.256*  (.100) |
| Years of Education | . 056**  (.018) | .058***  (.017) | .060***  (.017) | .059***  (.017) | .059***  (.017) | .051**  (.018) |
| Income (natural log) | .162***  (.047) | .149**  (.047) | .158***  (.047) | .158**  (.047) | .155***  (.047) | .147**  (.048) |
| Occupational Standing | .000  (.002) | .000  (.002) | .000  (.002) | .000  (.002) | .000  (.002) | .000  (.002) |
| Wealth ($10,00 units) | .002*  (.001) | .002*  (.001) | .002*  (.001) | .002*  (.001) | .002*  (.001) | .002*  (.001) |
| Child SES | .291  (.211) | .325  (.204) | .367  (.206) | .335  (.204) | .326  (.205) | .279  (.211) |
| Cognitive Ability | .261  (.264) |  |  |  |  | .204  (.271) |
| Trustworthy/Self-controlled |  | .394  (.207) |  |  |  | .363  (.206) |
| Valued Non-Cognitive Traits |  |  | .374  (.327) |  |  | .296  (.341) |
| Mastery |  |  |  | .390  (.231) |  | .251  (.279) |
| Self-esteem |  |  |  |  | .275  (.223) | .009  (.287) |
| Constant | 3.494*  (1.624) | 3.391*  (1.576) | 2.901  (1.580) | 2.872  (1.577) | 3.172*  (1.576 | 2.878  (1.651) |
| *p <.05; ** p<.01; ***p < .001  All equations control for age, and sex. Equation 6 additionally controls for child health, parents separated/divorced, parental problems with drugs or alcohol, and closeness to the mother at age 15-17. | | | | | | |

| Table S12. Negative Binomial Regression Equations with SES Dimensions Assessed Separately and in Absolute Metrics. Results test whether Cognitive and/or Non-Cognitive Traits Account for Race and SES Inequalities in Allostatic Load: Regression Coefficients (Standard Errors) N=400. | | | | | | |
| --- | --- | --- | --- | --- | --- | --- |
|  | Eq. 1 | Eq. 2 | Eq. 3 | Eq. 4 | Eq. 5 | Eq. 6 |
| Self- Identified Black Race | .249*  (.109) | .261**  (.101) | .264**  (.101) | .276**  (.101) | .288**  (.093) | .279*  (.116) |
| Years of Education | -.030  (.021) | -.032  (.020) | -.037  (.020) | -.033  (.020) | -.033  (.020) | -.034  (.021) |
| Income (natural log) | .033  (.053) | .046  (.054) | .029  (.051) | .032  (.053) | .033  (.053) | .046  (.054) |
| Occupational Standing | -.001  (.002) | -.001  (.002) | -.001  (.002) | -.001  (.002) | -.001  (.002) | -.001  (.002) |
| Wealth ($10,00 units) | -.003*  (.001) | -.003*  (.001) | .003*  (.001) | .003*  (.001) | .003*  (.001) | .003*  (.001) |
| Child SES | -.454  (.247) | -.507*  (.241) | -.441  (.242) | -.496*  (.242) | -.472  (.242) | -.425  (.255) |
| Cognitive Ability | -.193  (.315) |  |  |  |  | -.082  (.271) |
| Trustworthy/Self-controlled |  | -.331  (.221) |  |  |  | -.367  (.221) |
| Valued Non-Cognitive Traits |  |  | .673  (.369) |  |  | .881*  (.394) |
| Mastery |  |  |  | -.112  (.267) |  | -.085  (.337) |
| Self-esteem |  |  |  |  | -.156  (.270) | -.257  (.287) |
| Constant | 1.164  (1.840) | 1.181  (1.769) | .682  (1.806) | 1.591  (1.779) | 1.494*  (1.763) | .140  (1.934) |
| *p <.05; ** p<.01; ***p < .001  All equations control for age, and sex. Equation 6 additionally controls for child health, parents separated/divorced, parental problems with drugs or alcohol, and closeness to the mother at age 15-17. | | | | | | |

| Table S13 Multiple Regression Equations Assessing whether Cognitive and/or Non-Cognitive Traits Account for Race and SES Inequalities in Self-Rated Health with SES Coded as in Carter et al. Study: Regression Coefficients (Standard Errors), N=603 | | | | | | | | |
| --- | --- | --- | --- | --- | --- | --- | --- | --- |
|  | Eq. 1^1^ | Eq. 2^1^ | Eq. 3^1^ | Eq. 4^1^ | Eq. 5^1^ | Eq. 6^1^ | Eq. 7^1^ | Eq. 8^2^ |
| Self- Identified Black Race | -.444***  (.087) | -.346***  (.095) | -.408***  (.087) | -.465***  (.087) | -.445***  (.087) | -.485***  (.089) | -.368***  (.099) | -.359***  (.099) |
| Binary Adult Occupation | .265**  (.089) | .254**  (.089) | .248**  (.089) | .261***  (.089) | .249**  (.090) | .243**  (.090) | .223*  (.090) | .236**  (.092) |
| Binary Educational Attainment | .257*  (.091) | .195*  (.094) | .226*  (.091) | .246**  (.091) | .253**  (.091) | .244**  (.090) | .165  (.094) | .147  (.095) |
| Other Race/ethnicity | -.338*  (.141) | -.302*  (.141) | -.354*  (.141) | -.338*  (.141) | -.331*  (.141) | -.328*  (.141) | -.316*  (.141) | -.317*  (.140) |
| Binary Paternal Occupation at age 15 to 17 | .108  (.082) | .076  (.082) | .094  (.081) | .114  (.081) | .097  (.202) | .100  (.081) | .067  (.082) | .053  (.082) |
| Cognitive Ability |  | .626*  (.260) |  |  |  |  | .504^+^  (.263) | .540*  (.262) |
| Trustworthy/Self-controlled |  |  | .540*  (.216) |  |  |  | .477*  (.214) | .470*  (.219) |
| Valued Non-Cognitive Traits |  |  |  | .636*  (.325) |  |  | .456  (.328) | .490  (.335) |
| Mastery |  |  |  |  | .483*  (.235) |  | .197  (.277) | .119  (.278) |
| Self-esteem |  |  |  |  |  | .536*  (.239) | .222  (.284) | .210  (.285) |
| Constant | 6.246***  (1.510) | 6.923***  (1.533) | 6.246*  (1.517) | 5.659***  (1.546) | 5.844***  (1.479) | 6.098***  (1.511) | 6.143***  (1.587) | 5.650***  (1.596) |
| ^+^ P<.10; * p < .05; ** p< 01; ***p<.001  ^1^ Equations 1-7 include controls for age and sex.  ^2^Equation 8 includes controls for age, sex, child health, parental separation, parental substance use, and closeness to the mother as an adolescent. | | | | | | | | |
|  | | | | | | | | |

| Table S14. Regression Analyses Showing Significant Interactions Between Race and Cognitive Ability, Race and Trustworthy/self-controlled and Race and Mastery on Self-rated Health (N=603): Regression Coefficients (Standard Errors) | | | | | | | | | | |
| --- | --- | --- | --- | --- | --- | --- | --- | --- | --- | --- |
|  | Eq. 1 | Eq. 2 | Eq. 3 | Eq. 4 | Eq. 5 | Eq. 6 | Eq. 7 | Eq. 8 | Eq. 9 | Eq. 10 |
| Adult SES |  |  | 1.530***  (.203) | 1.495***  (.208) | 1.485***  (.207) | 1.491***  (.204) | 1.520***  (.201) | 1.477***  (.206) | 1.441***  (.212) | 1.472***  (.214) |
| Self-identified Black race | -.546***  (.082) | -.372***  (.092) | -.265**  (.089) | .200  (.219) | .431  (.283) | .280**  (.283) | .617  (.317) | -.295***  (.092) | 1.288^+^  (.658) | 1.340**  (.426) |
| Other race/ethnicity | -.373**  (.142) | -.278*  (.142) | -.379**  (.137) | -.477  (.392) | -.145  (.614) | -.377**  (.137) | 1.576  (.524) | -.371**  (.137) | 1.599  (.873) | 1.502  (.876) |
| Female | -.060  (.077) | -.053  (.074) | -.053  (.073) | -.033  (.074) | -.073  (.073) | -.063  (.073) | -.041  (.072) | -.038  (.074) | -.051  (.075) | -.066  (.075) |
| Age | -.051  (.031) | -.048  (.031) | -.049  (.029) | -.057^+^  (.030) | -.054^+^  (.029) | -.049^+^  (.030) | -.044  (.029) | -.052^+^  (.030) | -.053^+^  (.031) | -.052^+^  (.031) |
| Child SES |  | .804***  (.203) | .370^+^  (.202) | .259  (.210) | .329  (.202) | .399*  (.203) | .379^+^  (.199) | .355^+^  (.203) | .290  (.207) | .265  (.208) |
| Cognitive Ability |  |  |  | .764  (.339) |  |  |  |  | .478  (.338) | .501  (.338) |
| Cognitive * Black |  |  |  | -1.078*  (.483) |  |  |  |  | -.793  (.483) | -.703  (.481) |
| Cognitive * Other race |  |  |  | .298  (.793) |  |  |  |  | .566  (.789) | .558  (.792) |
| Trustworthy/Self-controlled |  |  |  |  | .874**  (.275) |  |  |  | .730**  (.280) | .721**  (.276) |
| Trustworthy * Black |  |  |  |  | -.946*  (.376) |  |  |  | -.766*  (.384) | -.745*  (.385) |
| Trustworthy * Other race |  |  |  |  | -.322  (.776) |  |  |  | -.489  (.759) | -.338  (.753) |
| Valued Non-Cognitive Traits |  |  |  |  |  | .471  (.292) |  |  | .352  (.302) | .359  (.305) |
| Mastery |  |  |  |  |  |  | 1.244***  (.293) |  | 1.043**  (.351) | .987**  (.333) |
| Mastery * Black |  |  |  |  |  |  | -1.369**  (.472) |  | -1.170*  (.483) | -1.208*  (.482) |
| Mastery * Other |  |  |  |  |  |  | -3.088***  (.802) |  | -2.922***  (.798) | -2,945***  (.799) |
| Self-esteem |  |  |  |  |  |  |  | .307  (.217) | -.035  (.274) | -.021  (.273) |
| Child health |  |  |  |  |  |  |  |  |  | -.502*  (.218) |
| Parents Separated/Divorced |  |  |  |  |  |  |  |  |  | .035  (.091) |
| Number of Siblings |  |  |  |  |  |  |  |  |  | .014  (.021) |
| Parental Problems with Drugs or Alcohol |  |  |  |  |  |  |  |  |  | -.178  (.109) |
| Close to Mother as an Adolescent |  |  |  |  |  |  |  |  |  | .348  (.256) |
| Constant | 6.246  (1.540) | 5.681  (1.529) | 5.151  (1.463) | 5.208  (1.503) | 4.760  (1.464) | 4.731  (1.491) | 4.090  (1.470) | 5.124  (1.467) | 3.646  (1.544) | 3.374  (1.567) |
| *p <.05; ** p<.01; ***p < .001 | | | | | | | | | | |

| Table S15. Complete Case Multiple Regression Equations Assessing whether Cognitive and/or Non-Cognitive Traits Account for Race and SES Inequalities in Self-Rated Health: Regression Coefficients (Standard Errors) | | | | | | | | | |
| --- | --- | --- | --- | --- | --- | --- | --- | --- | --- |
|  | Eq. 1 (N=603) | Eq. 2 (N=600) | Eq. 3 (N=598) | Eq. 4 (N=551) | Eq. 5 (N=453) | Eq. 6 (N=465) | Eq. 7 (N=466) | Eq. 8 (N=465) | Eq. 9 (N=381) |
| Self-identified Black race | -.546*** (.082) | -.364*** (.092) | -.261** (.090) | -.257** (.099) | -.325*** (.103) | -.426*** (.109) | -.419*** (.108) | -.431*** (.112) | -.479*** (.122) |
| Adult SES |  |  | 1.525*** (.204) | 1.538*** (.221) | 1.471*** (.234) | 1.523*** (.237) | 1.545*** (.237) | 1.509*** (.241) | 1.252*** (.257) |
| Other race/ethnicity | -.374** (.142) | -.279 (.143) | -.379** (.137) | -.360* (.140) | -.401** (.156) | -.320** (.145) | -.306* (.145) | -.309* (.147) | -.325* (.161) |
| Female | -.060 (.077) | -.059 (.077) | -.060 (.074) | -.010 (.078) | -.044 (.084) | .000 (.083) | -.006 (.083) | .019 (.085) | -.010 (.091) |
| Age | -.051 (.031) | -.047 (.031) | -.049 (.030) | -.041 (.038) | -.029 (.041) | -.028 (.043) | -.024 (.042) | -.028 (.043) | -.057 (.048) |
| Child SES |  | .800*** (.203) | .370 (.203) | .257 (.220) | .388 (.230) | .387 (.228) | .354 (.227) | .320 (.230) | .409 (.250) |
| Cognitive Ability |  |  |  | .308 (.266) |  |  |  |  | .257 (.316) |
| Trustworthy/Self-controlled |  |  |  |  | .415* (.201) |  |  |  | .607** (.216) |
| Valued Non-Cognitive Traits |  |  |  |  |  | .509 (.314) |  |  | .382 (.349) |
| Mastery |  |  |  |  |  |  | .423 (.233) |  | .219 (.296) |
| Self-esteem |  |  |  |  |  |  |  | .350 (.231) | .240 (.306) |
| Constant | 6.246*** (1.540) | 5.639*** (1.539) | 5.127*** (1.476) | 4.587* (1.898) | 3.869 (2.046) | 3.669 (2.134) | 3.627 (2.114) | 3.855 (2.133) | 4.494 (2.369) |

| Table S16. Complete Case Negative Binomial Regression Equations Assessing Whether Cognitive and/or Non-Cognitive Traits Account for Race and SES Inequalities in Allostatic Load: Regression Coefficients (Standard Errors) | | | | | | | | | |
| --- | --- | --- | --- | --- | --- | --- | --- | --- | --- |
|  | Eq. 1 (N=400) | Eq. 2 (N=400) | Eq. 3 (N=400) | Eq. 4 (N=372) | Eq. 5 (N=304) | Eq. 6 (N=323) | Eq. 7 (N=326) | Eq. 8 (N=326) | Eq. 9 (N=265) |
| Self-identified Black race | .456*** (.091) | .312** (.100) | .257* (.102) | .279* (.115) | .294* (.120) | .258* (.127) | .278* (.126) | .294* (.129) | .243 (.154) |
| Adult SES |  |  | -.616* (.242) | -.569* (.261) | -.546* .284 | -.839** (.286) | -.753** (.288) | -.751** (.288) | -.702* (.323) |
| Other race/ethnicity | .153 (.160) | .054 (.161) | .086 (.161) | .069 (.165) | .123 (.185) | .057 (.174) | .052 (.175) | .045 (.175) | .073 (.203) |
| Female | -.198* (.087) | -.219* (.086) | -.231** (.086) | -.277** (.090) | -.248* (.099) | -.312** (.099) | -.293** (.098) | -.302** (.099) | -.318** (.112) |
| Age | -.008 (.034) | -.011 (.034) | -.005 (.034) | -.021 (.044) | -.060 (.048) | -.036 (.049) | -.046 (.049) | -.043 (.049) | -.056 (.060) |
| Child SES |  | -.734** (.233) | -.563* (.241) | -.472 (.258) | -.552* (.280) | -.443 (.278) | -.470 (.278) | -.435 (.279) | -.394 (.330) |
| Cognitive Ability |  |  |  | -.256 (.320) |  |  |  |  | -.062 (.412) |
| Trustworthy/Self-controlled |  |  |  |  | -.336 (.239) |  |  |  | -.502 (.275) |
| Valued Non-Cognitive Traits |  |  |  |  |  | .618 (.392) |  |  | .878 (.464) |
| Mastery |  |  |  |  |  |  | -.174 (.267) |  | -.208 (.368) |
| Self-esteem |  |  |  |  |  |  |  | -.210 (.278) | -.092 (.404) |
| Constant | 1.174 (1.691) | 1.713 (1.675) | 1.671 (1.661) | 2.553 (2.153) | 4.640 (2.335) | 2.800 (2.464) | 3.849 (2.422) | 3.744 (2.411) | 4.049 (2.940) |

**Figure S2.** Graphed Interactions Between Race and Traits on Self-Rated Health Adjusting for Age, Sex, Childhood SES, and Adult SES.

Panel A: Cognitive Ability

Panel B: Dependable Trustworthy

**Panel C: Mastery**
